# *Plasmopara viticola *effector PvRXLR131 suppresses plant immunity by targeting plant receptor‐like kinase inhibitor BKI1

**DOI:** 10.1111/mpp.12790

**Published:** 2019-04-04

**Authors:** Xia Lan, Yunxiao Liu, Shiren Song, Ling Yin, Jiang Xiang, Junjie Qu, Jiang Lu

**Affiliations:** ^1^ College of Food Science and Nutritional Engineering China Agricultural University Beijing China; ^2^ Center for Viticulture and Enology, School of Agriculture and Biology Shanghai Jiao Tong University Shanghai China; ^3^ Guangxi Crop Genetic Improvement and Biotechnology Laboratory Guangxi Academy of Agricultural Sciences Nanning China

**Keywords:** BKI1, brassinosteroid, downy mildew, ERECTA, *Plasmopara viticola*, RXLR effector

## Abstract

The grapevine downy mildew pathogen *Plasmopara viticola* secretes a set of RXLR effectors (PvRXLRs) to overcome host immunity and facilitate infection, but how these effectors function is unclear. Here, the biological function of PvRXLR131 was investigated via heterologous expression. Constitutive expression of PvRXLR131 in *Colletotrichum gloeosporioides* significantly enhanced its pathogenicity on grapevine leaves. Constitutive expression of PvRXLR131 in *Arabidopsis* promoted *Pseudomonas* *syringae* DC3000 and *P.* *syringae* DC3000 (*hrcC^‐^*) growth as well as suppressed defence‐related callose deposition. Transient expression of PvRXLR131 in *Nicotiana benthamiana* leaves could also suppress different elicitor‐triggered cell death and inhibit plant resistance to *Phytophthora capsici*. Further analysis revealed that PvRXLR131 interacted with host *Vitis vinifera* BRI1 kinase inhibitor 1 (VvBKI1), and its homologues in *N. benthamiana* (NbBKI1) and *Arabidopsis* (AtBKI1). Moreover, bimolecular fluorescence complementation analysis revealed that PvRXLR131 interacted with VvBKI1 in the plasma membrane. Deletion assays showed that the C‐terminus of PvRXLR131 was responsible for the interaction and mutation assays showed that phosphorylation of a conserved tyrosine residue in BKI1s disrupted the interaction. BKI1 was a receptor inhibitor of growth‐ and defence‐related brassinosteroid (BR) and ERECTA (ER) signalling. When silencing of *NbBKI1* in *N. benthamiana*, the virulence function of PvRXLR131 was eliminated, demonstrating that the effector activity is mediated by BKI1. Moreover, *PvRXLR131*‐transgenic plants displayed BKI1‐overexpression dwarf phenotypes and suppressed BR and ER signalling. These physiological and genetic data clearly demonstrate that BKI1 is a virulence target of PvRXLR131. We propose that *P. viticola* secretes PvRXLR131 to target BKI1 as a strategy for promoting infection.

## Introduction

In nature, pathogens attack host plants to obtain nutrients and complete their life cycle. Plants, in turn, attempt to fight against these pathogens to ensure survival. Plant‐pathogen interactions involve detection of pathogens and activation of immunity in plants, as well as evasion of detection and suppression of immunity by pathogens. Plants recognize broadly conserved pathogen/microbe‐associated molecular patterns (PAMPs/MAMPs) through pattern recognition receptors (PRRs) located in the plasma membrane, thereby activating the first layer of innate immune system termed PAMP/MAMP‐triggered immunity (PTI; Jones and Dangl, 2006). PTI is generally effective against non‐adapted pathogens (Dodds and Rathjen, 2010). Pathogens, in turn, suppress PTI by secreting effector proteins, which function in plant apoplast (apoplastic effectors) or cytoplasm (cytoplasmic effectors) (Jones and Dangl, 2006; Kamoun, 2006). As a counter measure, plants have evolved intracellular resistance (R) proteins to specifically detect some effectors directly or indirectly, resulting in activation of the second layer of innate immune system called effector‐triggered immunity (ETI; Jones and Dangl, 2006). ETI is a strong immune response and typically accompanied by a hypersensitive response (HR) that restricts pathogen invasion (Greenberg and Yao, 2004). Under natural selection pressure, pathogens evolve new effectors to suppress ETI or shed and diversify recognized effector genes to avoid ETI. Conversely, plants develop new *R *genes so that ETI can be triggered again. These continuous co‐evolutionary arms race between pathogens and plants secure their survival and propagation (Dodds and Rathjen, 2010; Jones and Dangl, 2006).

Oomycetes are eukaryotic microorganisms with features resembling filamentous fungi, but exhibits phylogenetic relationships with diatoms and brown algae in stramenopiles (Beakes *et al*., 2012; Sekimoto *et al*., 2008). Some oomycetes are notorious pathogens that have caused great damage to agriculture, including obligate biotrophs causing downy mildew (*Plasmopara viticola*, *Hyaloperonospora arabidopsidis*), hemibiotrophs in *Phytophthora* (*Phytophthora infestans*, *Phytophthora sojae*, *Phytophthora capsici*), and necrotrophs in *Pythium* (*Pythium ultimum*) (Kamoun *et al*., 2015). Similar to other pathogens, oomycetes also employ an array of effectors to modify plant metabolism to their benefit and promote infection. Apoplastic effectors include enzyme inhibitors, small cysteine‐rich proteins and Nep1‐like proteins that interact with the extracellular targets or surface receptors of the host (Kamoun, 2006). Compared with the functions of apoplastic effectors, the functions of most cytoplasmic effectors are less understood because the lack of sequence similarity to known proteins makes it difficult to predict their functions and mechanisms (Li *et al*., 2016b; Liu *et al*., 2018). RXLR (arginine‐any amino acid‐leucine‐arginine) and CRN (crinkling‐ and necrosis‐inducing) families are the two most important categories of oomycete cytoplasmic effectors. RXLR effectors are modular proteins in which the N‐terminus carries a signal peptide (SP), followed by a RXLR/RXLR‐like motif (some also possess an EER (glutamic acid‐glutamic acid‐arginine) motif downstream), and finally a C‐terminal functional region for effector activity (Bhattacharjee *et al*., 2006; Kamoun, 2006; Whisson *et al*., 2007). The RXLR motif was considered to responsible for effector entering the host cell, but this biological function is still controversial (Ellis and Dodds, 2011; Wawra *et al*., 2013, 2017). To date, at least 37 oomycete species have undergone genome sequencing, based on which a large number of effectors was predicted (Mccarthy and Fitzpatrick, 2017). Unlike CRN effectors that are ubiquitous in different oomycete species, RXLR effectors are mainly found in *Phytophthora* and downy mildew species, with few or no in other oomycete lineages, suggesting that RXLR effectors play key roles in virulence in *Phytophthora* and downy mildew species (Anderson *et al*., 2015).

Functional genomic studies have been carried out to characterize RXLR effectors. Notably, suppression of host immunity seems to be a major function of most effectors, as was demonstrated in the model plant *Nicotiana benthamiana *by monitoring their ability to suppress elicitor‐triggered cell death or to enhance pathogen leaf colonisation and in the bacteria *Pseudomonas syringae* by assessing their ability to promote pathogen pathogenicity (Fabro *et al*., 2011; Liu *et al*., 2018; Wang *et al*., 2011, 2019; Xiang *et al*., 2016). RXLR effectors target diverse pathways within the host cell to suppress plant immunity, such as preventing secretion of defence‐related proteases (Bozkurt *et al*., 2011), reducing the accumulation of reactive oxygen species around invasion sites (Dong *et al*., 2011), perturbing mitogen‐activated protein kinase (MAPK) pathways (King *et al*., 2014), supporting or promoting the activity of negative immune regulators (Boevink *et al*., 2016; Murphy *et al*., 2018), suppressing RNA silencing and reducing accumulation of small RNAs (Qiao *et al*., 2013, 2015), and reprograming pre‐mRNA splicing (Huang *et al*., 2017). Although accumulating evidence shows that pathogenic bacteria and fungi deploy effectors to manipulate phytohormone pathways to defeat immunity (Kazan and Lyons, 2014; Ma and Ma, 2016), a few RXLR effectors were identified to connect to hormone signalling pathways. Caillaud *et al*. (2013) reported that the *H. arabidopsidis* effector HaRXL44 shifted defence transcription from salicylic acid (SA) to jasmonic acid/ethylene (JA/ET) signalling and enhanced host susceptibility to biotrophs. The *Phytophthora parasitica* effector PSE1 interferes with plant auxin accumulation to promote infection (Evangelisti *et al*., 2013). The *P. infestans *effector Avr2 up‐regulates a brassinosteroid (BR)‐responsive bHLH transcription factor through antagonistic crosstalk between BR signalling and innate immunity to suppress immunity indirectly (Turnbull *et al*., 2017).

Downy mildew is a devastating disease of grapevines and causes great economic losses to the grape industry throughout the world (Gessler *et al*., 2011). The causal agent of grapevine downy mildew is *P. viticola* that typically requires living host cells to complete infection cycle (Viennot‐Bourgin, 1949). At least 100 RXLR effectors (PvRXLR) have been predicted from the *P. viticola* ‘JL‐7‐2’ genome, 18 of which showed more than 30% amino acid sequence identity to RXLR effectors from other oomycete species (Yin *et al*., 2017). Poor conservation exists in RXLR effectors across different oomycetes, and it has been postulated that amongst oomycete conserved RXLR effectors exist ‘core’ pathogenicity genes that target cellular processes shared by diverse plant species (Anderson *et al*., 2012; Baxter *et al*., 2010; Deb *et al*., 2018). Therefore, unravelling the molecular functions of these effectors is central to understand oomycete pathogenesis. Accordingly, in this study, we conducted a functional analysis of the conserved effector PvRXLR131 to explore its roles in pathogenicity and modulating plant physiology, search for its potential host targets, and elucidate the molecular mechanisms underlying pathogenicity. This is the first study to report that oomycete effector targets inhibitor of receptor‐like kinases to promote infection. Our findings present valuable information for further understanding of grapevine/*P. viticola* interactions and provide new insight into the mechanisms underlying oomycete virulence.

## Results

### PvRXLR131 is an evolutionarily conserved RXLR effector

We were interested in conserved RXLR effectors amongst different oomycetes, because effector homologues might contribute to a conserved and general virulence function in different hosts. In the present study, we focused on *P. viticola *effector PvRXLR131. *PvRXLR131 *encodes a protein of 158 amino acids. The BLASTP search against the National Center for Biotechnology Information (NCBI) database indicated that PvRXLR131 showed moderate identity to RXLR effectors from other oomycetes including *P. parasitica *(38%), *P. infestans *(34%), *P. sojae *(35%), and *Plasmopara halstedii *(36%) (Table 1). Each effector contained a predicted SP, followed by an RXLR/RXLR‐like motif and an EER motif (Table 1; Fig. S1, see Supporting Information). Besides these, no other functional domains were predicted.

**Table 1:** Bioinformatic analysis of PvRXLR131 and its homologues.

Description	Accession number	Species	Gene name	Length*	Signal peptide		RXLR‐dEER motif	RXLR position†	Identity (%)‡
					Length*	S‐score			
Predicted RXLR effector	Pv05707	*Plasmopara viticola*	PvRXLR131	158	20	0.960	RDLDGSTTSMSVNVDDEER	39	100
Hypothetical protein	XP_008899021.1	*Phytophthora parasitica*	PPTG_06733	175	20	0.977	RNLKGSSTTTAEEEER	42	38
Secreted RXLR effector peptide protein	XP_002898683.1	*Phytophthora infestans*	PITG_15235	183	20	0.984	RRLKGAITATEGAVAEDEER	40	34
RXLR‐like protein	XP_024572649.	*Plasmopara halstedii*	PHALS_02189	503	19	0.986	NSLRSSIKTKDEER	49	36
Avh263 (RXLR effector)	AEK81067.1	*Phytophthora sojae*	PsAvh263	176	21	0.977	RSLRSSVTTQDAEAEER	26	35

* Length in amino acids.

† Position counting from the N terminus.

‡ Compared with PvRXLR131.

### PvRXLR131 is induced during infection

As *PvRXLR131* was predicted in *P. viticola* ‘JL‐7‐2’ genome, it is necessary to determine whether this gene is expressed during infection. The susceptible grapevine (*Vitis vinifera* ‘Thompson Seedless’) leaves were inoculated with *P. viticola* ‘JL‐7‐2’. The transcript level of *PvRXLR131 *was measured at different time points post‐inoculation (0, 6, 12, 24, 36, 48, 60, 72, 96 and 120 h post‐inoculation [hpi]). Results showed that the expression of* PvRXLR131* was negligible before 12 hpi, but it increased at 24 hpi and peaked at 36 hpi (130‐fold), and then declined during the subsequent stages of the interaction (Fig. S2A, see Supporting Information). The growth of *P. viticola* in plant was also monitored during the experiment (Fig. S2B, see Supporting Information). The expression pattern of *PvRXLR131* was similar to that of the second group of *PvRXLRs *(Xiang *et al*., 2016), and these genes were highly expressed during the earlier infection stages. In addition, *PvRXLR131* was successfully detected in 48 *P. viticola *isolates (Fig. S2C, see Supporting Information) collected from eight grapevine‐growing provinces in China and from grapevine cultivars with different resistance levels (Li *et al*., 2016a). Thus, PvRXLR131 might play a role in the *P. viticola*–grapevine interaction.

### PvRXLR131 contains a functional signal peptide

**Figure 1:**
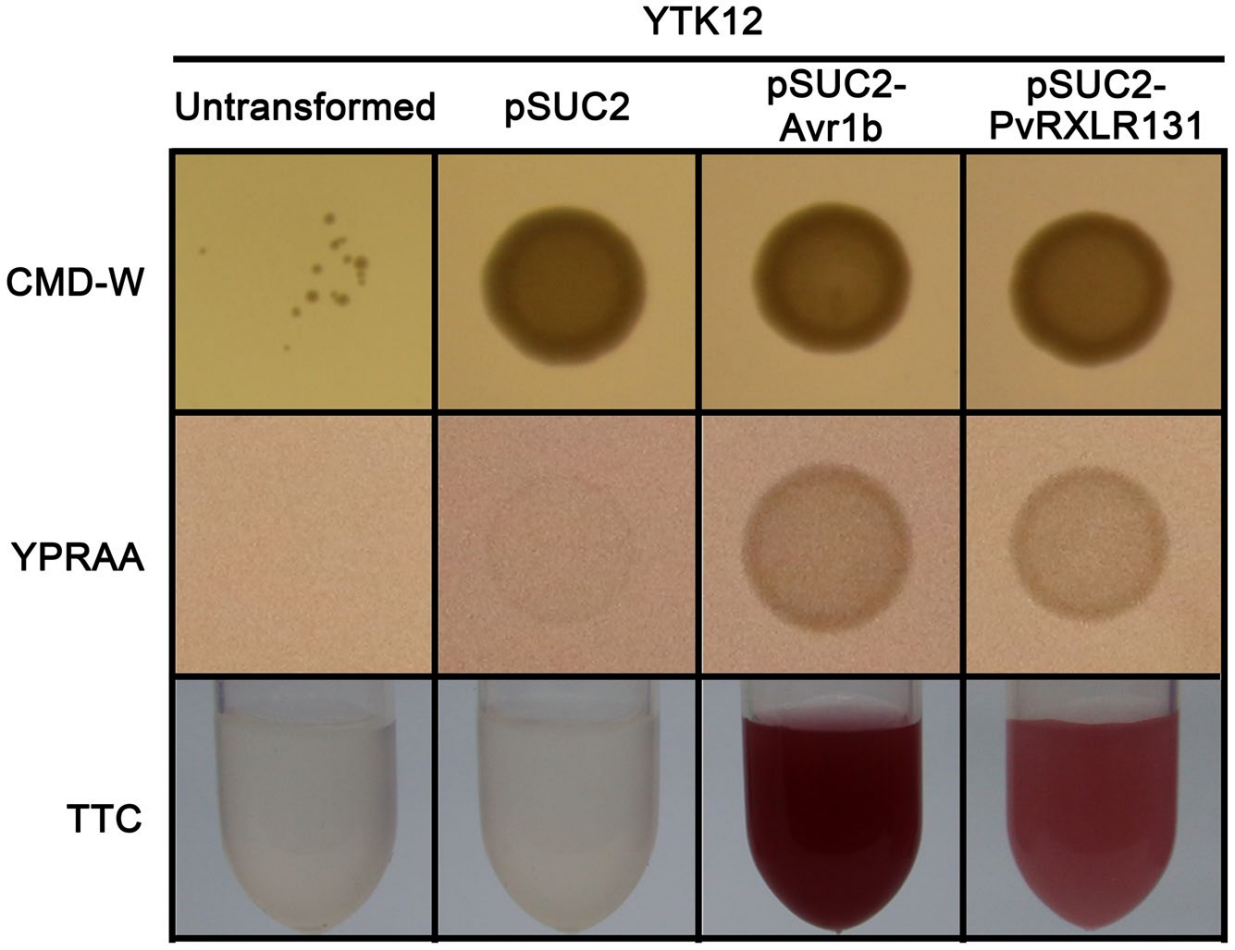
Functional validation of the putative PvRXLR131 signal peptide (SP). The pSUC2 empty vector and the pSUC2‐Avr1b and pSUC2‐PvRXLR131 recombinant plasmids were transformed into the invertase‐deficient yeast strain YTK12. CMD‐W medium was used for screening positive transformants. YPRAA medium and TTC were used to analyse invertase secretion, only clones carrying a functional SP can grow on YPRAA and elicit the red colour change in TTC.

The N‐terminal 20 amino acids of PvRXLR131 were predicted to be SP with a score value of 0.96 (Table 1), suggesting a high probability of secretion. To validate whether this predicted SP was functional, we performed yeast signal sequence trap (SST) assay based on the requirement of secreted invertase for yeast growth on medium with only sucrose or raffinose as carbon source (Jacobs *et al*., 1997; Oh *et al*., 2009). When the predicted SP was introduced into the pSUC2 vector to create in‐frame fusions with invertase and transformed into the invertase‐deficient yeast strain YTK12, the YTK12 carrying pSUC2‐PvRXLR131 or pSUC2‐Avr1b (positive control) had the ability to grow on YPRAA medium (raffinose as the only carbon source), while YTK12 harbouring an pSUC2 empty vector and untransformed YTK12 were unable to grow (Fig. 1). The secreted invertase can hydrolyse sucrose into monosaccharides, which transform colourless 2,3,5‐triphenyltetrazolium chloride (TTC) into red coloured 1,3,5‐triphenylformazan. This colourimetric test was used to confirm the secretion further. As expected, TTC‐treated suspensions of untransformed YTK12 and YTK12 carrying the empty vector remained colourless, whereas that of PvRXLR131 and Avr1b transformants appeared red in colour (Fig. 1), suggesting that PvRXLR131 SP was functional.

### PvRXLR131 is required for pathogen virulence

**Figure 2:**
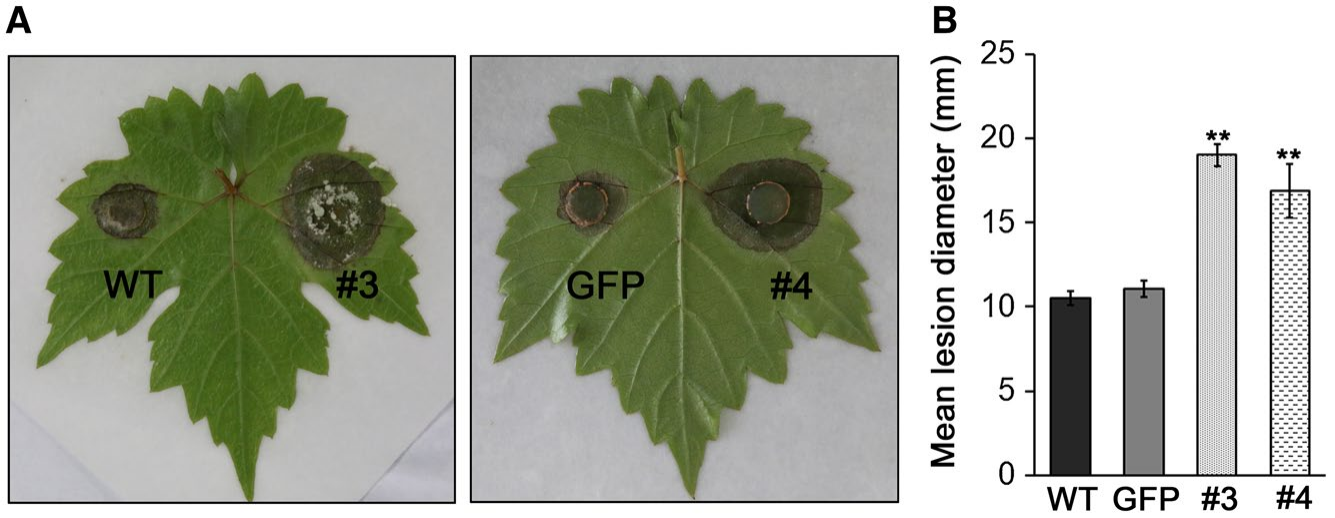
PvRXLR131 enhances *Colletotrichum gloeosporioides* pathogenicity. Disease lesions on grapevine leaves caused by wild type (WT), *GFP*‐transgenic and *PvRXLR131‐*transgenic (#3, #4) *C. gloeosporioides* are shown (A). Photographs were taken 6 days after inoculation. Corresponding lesion diameters were measured (B). The data represent means ± standard deviations (SDs) from three biological replicates; asterisks indicate significant differences from WT (**P < 0.01; LSD).

The most effective method to investigate the role of PvRXLR131 during pathogen infection is to overexpress or knock out this gene in *P. viticola*. Unfortunately, this method is difficult because the obligate biotrophic lifestyle of *P. viticola* limits *in vivo* genetic engineering. *Colletotrichum* *gloeosporioides*, the causal agent of grape ripe rot, is a semi‐biotrophic fungus and is easy to manipulate genetically. This pathogen was therefore selected for evaluating the role of PvRXLR131 in pathogenicity. Full‐length *PvRXLR131* and control *GFP* were introduced into *C. gloeosporioides*, respectively, driven by the ToxA promoter. Grapevine leaves were inoculated with two lines stably expressing PvRXLR131(#3 and #4), one line stably expressing GFP, and the wild type (WT) (Fig. S3, see Supporting Information). In comparison to the *GFP* and WT controls, the two *PvRXLR131*‐transgenic lines grew faster and developed disease lesions more than 1.5 times larger (Fig. 2A and B). This result clearly indicated that PvRXLR131 enhanced the pathogenicity of *C. gloeosporioides*, implying that this effector might play an important role in virulence during *P. viticola* infection.

### *PvRXLR131*‐transgenic plants exhibit dwarf phenotype

**Figure 3:**
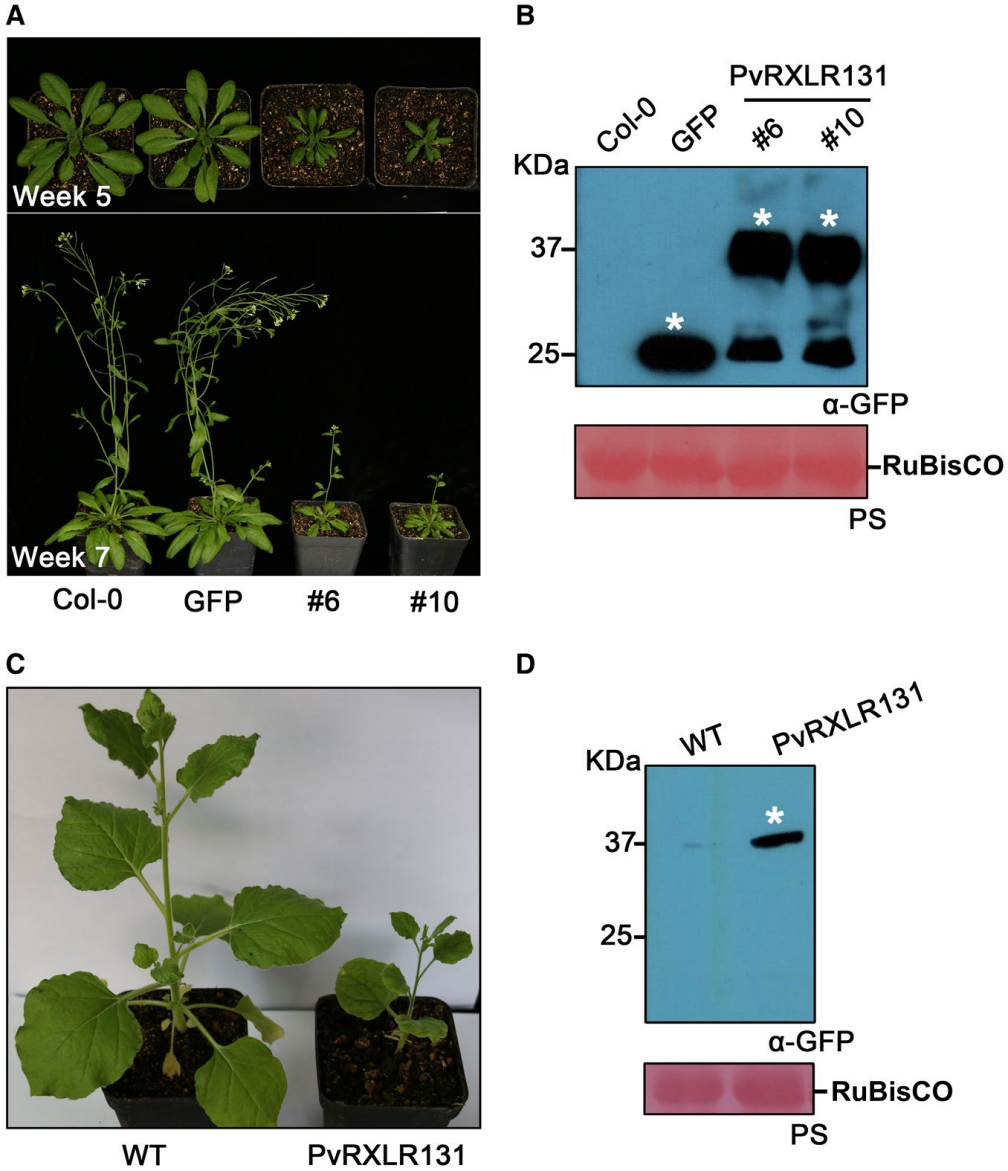
*PvRXLR131*‐transgenic plants display dwarf phenotype. (A) Phenotype analysis of *Arabidopsis*. Phenotypes of *PvRXLR131*‐transgenic plants (#6, #10) were compared with those of *GFP*‐transgenic and Col‐0 plants. Protein expression are shown (B). (C) Phenotype analysis of *Nicotiana benthamiana*. *PvRXLR131*‐transgenic *N. benthamiana* displays a dwarf phenotype compared with wild type (WT). Protein expression are indicated (D). Protein was detected by Western blotting. White asterisks indicate corresponding protein. Ponceau‐S (PS) stained RuBisCO large subunit (rbcL) serves as a loading control.

Transgenic *Arabidopsis thaliana *ecotype Columbia (Col‐0) stably overexpressing PvRXLR131 with a GFP tag driven by 35S promoter were produced. Two homozygotes (referred to hereafter as #6 and #10) were obtained by self‐crossing, and the expression of PvRXLR131‐GFP was confirmed by Western blotting (Fig. 3B). Notably, we found that transgenic *Arabidopsis* overexpressing PvRXLR131‐GFP displayed a dwarf phenotype, with smaller rosette, reduced stature, shorter petiole, delayed flowering and fewer inflorescences when compared with *GFP *and Col‐0 controls (Figs 3A and S4, see Supporting Information). In addition, overexpressed PvRXLR131‐GFP in *N. benthamiana *resulted in dwarf plants as well (Fig. 3C and D). These data demonstrated that the accumulation of PvRXLR131 significantly affected the growth and development of *Arabidopsis* and *N. benthamiana*, indicating that PvRXLR131 might target a protein involved in plant development and it was conserved in different plant species.

### PvRXLR131 suppresses plant innate immunity

**Figure 4:**
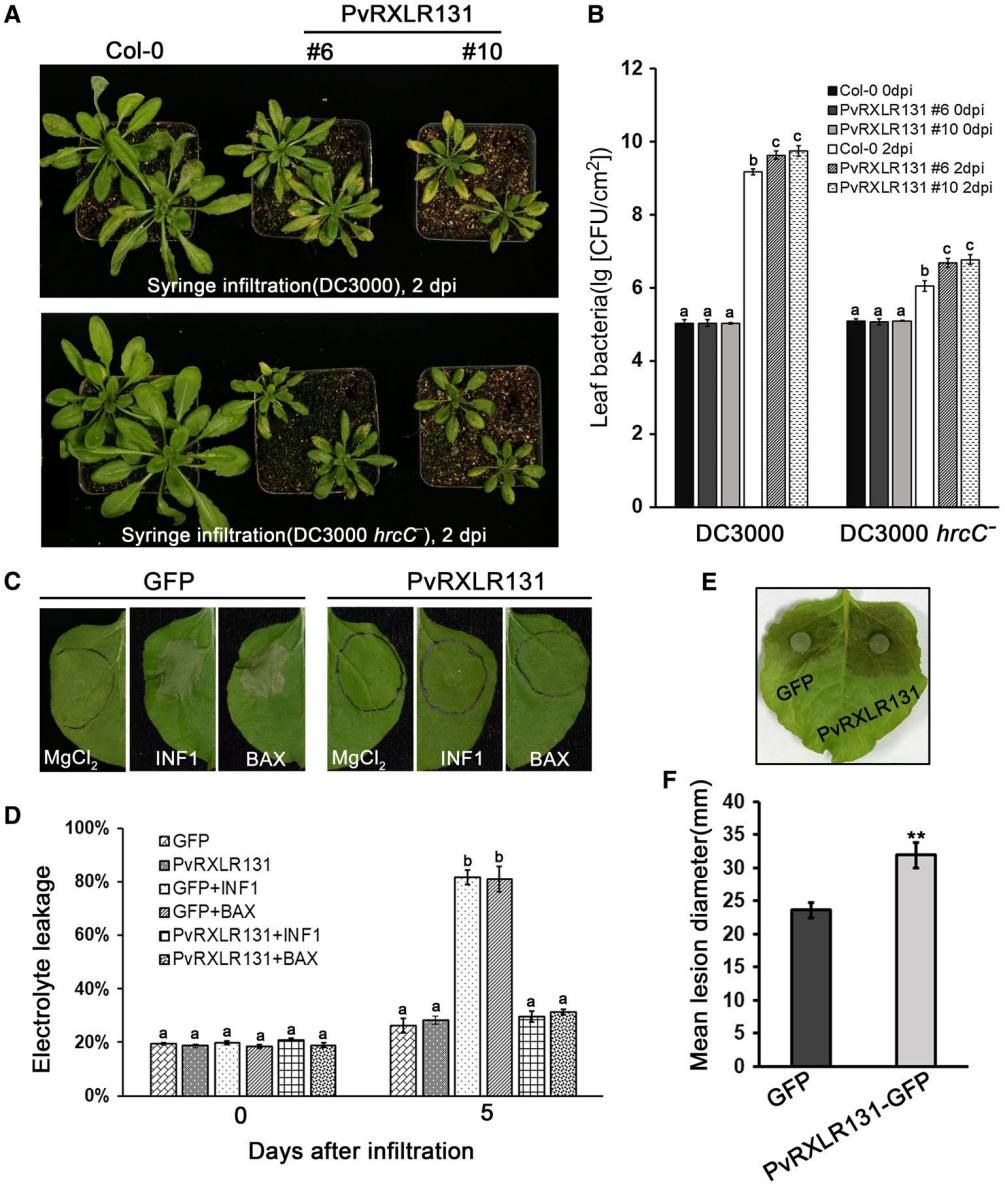
PvRXLR131 suppresses plant innate immunity. (A) PvRXLR131 impairs host resistance to *Pseudomonas syringae* pathovar tomato DC3000 and the mutant strain DC3000 (*hrcC−*)*. *Two strains were infiltrated into *Arabidopsis* leaves and phenotypes were scored at 2 dpi. Representative images are shown. Quantitation of bacterial amount are labelled (B). Each data point represents means ± standard deviations (SDs) from three biological replicates; values with different letters show significant differences between mean values at *P* = 0.05 (LSD). (C) PvRXLR131 suppresses cell death triggered by two elicitors in *Nicotiana benthamiana*. *Agrobacterium *GV3101 carrying the pGR106‐PvRXLR131 and pGR106‐GFP constructs were simultaneously infiltrated into *N. benthamiana* leaves; GV3101 containing pGR106‐elicitor was infiltrated at the first infiltration region after 24 h. MgCl_2 _(10 mM) was infiltrated as the negative controls. Cell death was scored 5 days after the second infiltration. Quantification of cell death by electrolyte leakage are shown (D). The data represent means ± standard deviations (SDs) from three biological replicates; values with different letters show significant differences between mean values at *P* = 0.01 (LSD). (E) PvRXLR131 accumulation increases *Phytophthora capsici* growth on *N. benthamiana*. PvRXLR131‐GFP and GFP were transiently simultaneously expressed in *N. benthamiana* leaves; leaves were detached at 36 h, followed by inoculation with *P. capsici *discs on the abaxial side of the leaves. Disease development was evaluated at 3 dpi and growth area was plotted as average lesion diameter (F). Each data point represents means ± standard deviations (SDs) from three biological replicates; asterisks indicate significant differences from GFP control (**P < 0.01, Student's *t*‐test).

To evaluate the virulence function of PvRXLR131 *in planta*, *PvRXLR131*‐transgenic *Arabidopsis* and Col‐0 were infiltrated with *P. syringae* pathovar tomato DC3000 and its mutant strain DC3000 (*hrcC^−^*). The latter lacks a functional type III secretion system that is responsible for effector injection into plant cells and is almost non‐pathogenic (Yuan and He, 1996). *PvRXLR131*‐transgenic plants appeared more susceptible to both DC3000 and DC3000 (*hrcC^−^*), exhibiting chlorosis and water‐soaked infected leaves, whereas leaves of Col‐0 remained green with only slightly wilting (Fig. 4A). Population of DC3000 and DC3000 (*hrcC^−^*) in *PvRXLR131*‐transgenic plants was approximately three‐ and five‐fold greater than those in Col‐0 despite having nearly the same initial bacterial population (Fig. 4B). Besides, callose deposition, an output of PTI, was suppressed by ~30% in *PvRXLR131*‐transgenic plants when treated with PAMP flg22 (a 22‐amino acid bacterial flagellin peptide) (Fig. S5, see Supporting Information). These results indicated that PvRXLR131 attenuated the resistance of *Arabidopsis *to *P. syringae* with diminished callose deposition.

Next, we tested the ability of PvRXLR131 in suppressing elicitor‐triggered cell death in *N. benthamiana.* The elicitors included pro‐apoptotic protein BAX from mouse (Lacomme and Cruz 1999) and the elicitin INF1 from *P. infestans* (Kamoun *et al*., 1998). When PvRXLR131 and GFP were transiently expressed in *N. benthamiana *leaves followed by challenging with the elicitors. GFP‐expressing regions treated with BAX or INF1 appeared noticeable necrosis, whereas PvRXLR131‐expressing regions with the same treatments did not show cell death at all (Fig. 4C). Protein expression was confirmed by Western blotting (Fig. S6A, see Supporting Information). Electrolyte leakage assay was performed to quantify cell death. Consistent with the phenotype observations, electrolyte leakage from *N. benthamiana* leaves with PvRXLR131 accumulation was significantly lower than that from the GFP controls (Fig. 4D). These results demonstrated that PvRXLR131 acted as a suppressor of defence‐related cell death in *N. benthamiana*. Furthermore, overexpression of PvRXLR131‐GFP in *N. benthamiana* leaves significantly increased the growth of *P. capsici *(lesion diameter, PvRXLR131, 31.9 ± 1.99 mm; GFP, 23.6 ± 1.11 mm) (Figs 4E, F and S6B, see Supporting Information), suggesting that PvRXLR131 caused enhanced susceptibility of *N. benthamiana* to oomycetes.

These findings demonstrated that PvRXLR131 could attenuate plant immunity.

### PvRXLR131 interacts with plant BKI1 in plasma membrane

To identify the grapevine interactors of PvRXLR131, Y2H screening with a *P. viticola*‐infected grapevine cDNA library was performed using mature PvRXLR131 as a bait. In three independent screenings, grapevine cDNA fragments from one gene were consistently captured. This gene was annotated as *V. vinifera* BRI1 kinase inhibitor 1, and we designated this gene as *VvBKI1*. The encoding protein VvBKI1 was 315 amino acids in length. Sequence analysis confirmed that the VvBKI1 was homologous to AtBKI1 in *Arabidopsis*, a protein involved in growth‐ and defence‐related BR and ERECTA (ER) signalling pathways (Llorente *et al*., 2005; Wang *et al*., 2017).

**Figure 5:**
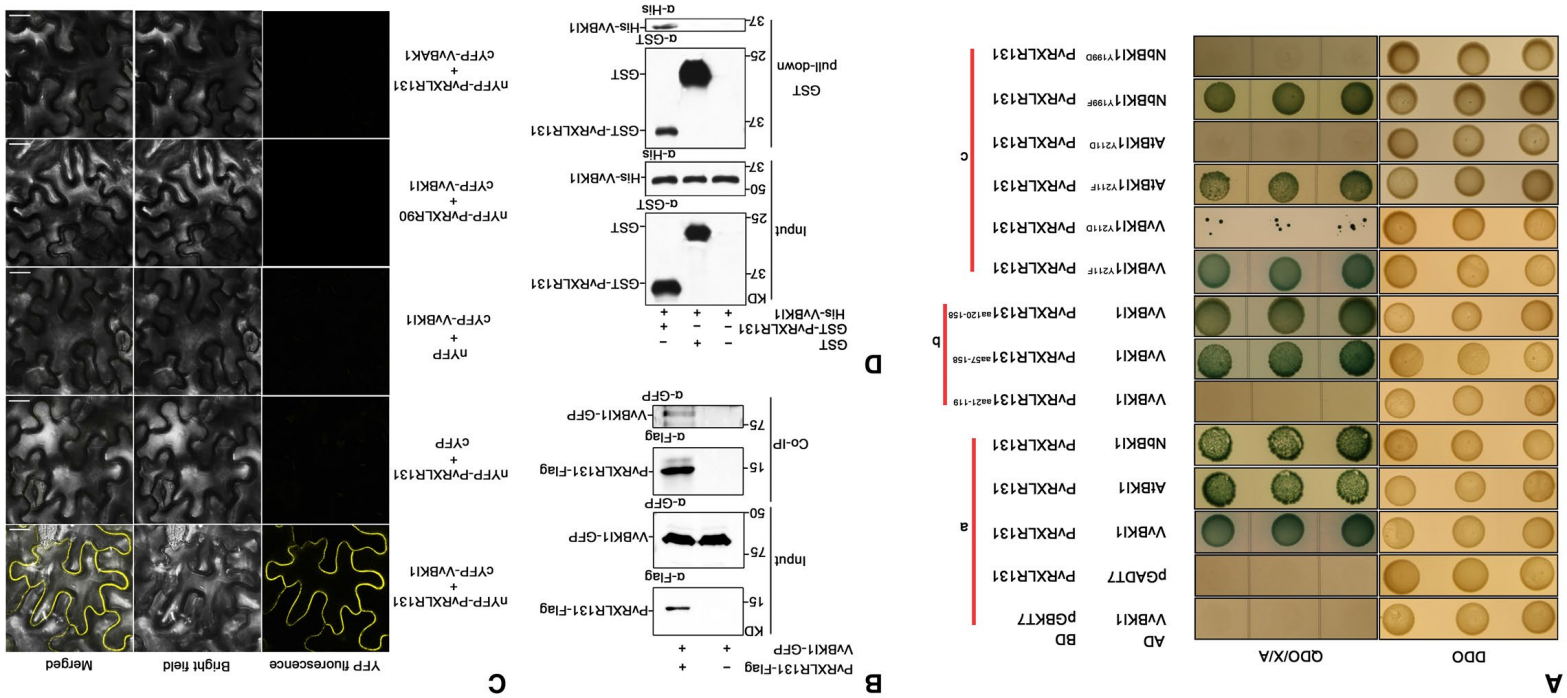
Confirmation of the interaction between PvRXLR131 and VvBKI1. (A) Y2H analysis illustrates the interaction between BKI1 and PvRXLR131. Yeast strain Y2H Gold containing both prey (AD) and bait (BD) can grow on DDO non‐selective medium. Colonies on DDO were drip inoculated on QDO selective medium supplemented with X‐α‐Gal and aureobasidin A (QDO/X/A); strains harbouring two interacting proteins can grow on QDO/X/A and turn blue. The empty vector pGBKT7(BD) or pGADT7(AD) was used as control. Group a demonstrated that PvRXLR131 interacted with VvBKI1, AtBKI1 and NbBKI1. The C‐terminal effector domain of PvRXLR131 was responsible for the interaction between VvBKI1 and PvRXLR131 (Group b). Mutagenesis of BKI1s at a conserved tyrosine site (VvBKI1^Y211^, AtBKI1 ^Y211^ and NbBKI1^Y199^) into aspartic acid disrupted the interaction with PvRXLR131, while phenylalanine mutations did not (Group c). (B) Confirmation of the interaction using co‐immunoprecipitation (Co‐IP). VvBKI1‐GFP was transiently expressed alone or with PvRXLR131‐Flag in *Nicotiana benthamiana*. Total proteins were extracted and immunoprecipitated with anti‐Flag antibody. Extracted proteins and the bound protein were detected using immunoblot with the indicated antibodies. (C) Confirmation of the interaction using BiFC. The C‐terminus of YFP (cYFP) was fused to the N‐terminus of VvBKI1 and VvBAK1 (control), and the N‐terminus of YFP (nYFP) was fused to the N‐terminus of PvRXLR131 and PvRXLR90 (control); both of the indicated recombinant proteins were transiently co‐expressed in *N. benthamiana*. Fluorescence signals of YFP were observed at 2 dpi using confocal microscopy. Bars = 20 µm. (D) Confirmation of the interaction using GST pull‐down. His‐VvBKI1, GST‐PvRXLR131 and GST were affinity purified. GST pull‐down assay was performed for each combination as indicated. The amount of bound protein His‐VvBKI1 was analysed using anti‐His immunoblot.

To confirm the interaction between PvRXLR131 and VvBKI1, four independent assays were performed. First, we used full‐length cDNA of *VvBKI1* to conduct yeast two‐hybrid (Y2H) assays. VvBKI1 showed strong interaction with PvRXLR131 and colonies of yeast strain Y2H Gold expressing both BD‐PvRXLR131 and AD‐VvBKI1 grew rapidly on the stringent quadruple dropout medium supplemented with X‐α‐Gal and Aureobasidin A (QDO/X/A) plates (Fig. 5A). We then performed co‐immunoprecipitation (co‐IP) experiments. VvBKI1‐GFP was transiently expressed alone or co‐expressed with PvRXLR131‐Flag in *N. benthamiana*. Total proteins were isolated and incubated with anti‐Flag beads. VvBKI1‐GFP was only detectable in co‐IP product from protein extracts containing VvBKI1‐GFP and PvRXLR131‐Flag (Fig. 5B). We also used bimolecular fluorescence complementation (BiFC) to monitor their interaction site in plant cells. Another *P. viticola* effector PvRXLR90 that has been confirmed to target plant plasma membrane (Liu *et al*., 2018) and the *V. vinifera* protein BAK1 (VvBAK1), which is a well‐known plasma membrane‐localized protein were used as the controls (Cheng *et al*., 2011). The results showed that the YFP signal of PvRXLR131 and VvBKI1 chimeras was successfully detected and exclusively accumulated in the plasma membrane, while no YFP signal was detected in all controls (Fig. 5C). Finally, GST pull‐down assays were applied to verify the direct interaction between PvRXLR131 and VvBKI1 *in vitro*. GST, GST‐PvRXLR131, and His‐VvBKI1 were expressed in prokaryotic systems, and different combinations of proteins were mixed. Of all the combinations tested, only GST‐PvRXLR131 co‐precipitated His‐VvBKI1 (Fig. 5D), demonstrating that PvRXLR131 directly interacted with VvBKI1 *in vitro*. In summary, the above results all agreed that PvRXLR131 physically interacted with VvBKI1.

To determine which part of PvRXLR131 interacted with VvBKI1, truncated constructs of PvRXLR131 were generated (Fig. S7A, see Supporting Information). Y2H analysis demonstrated that the C‐terminus (PvRXLR131^aa57‐158^, PvRXLR131^aa120‐158^) but not the N‐terminus (PvRXLR131^aa21‐119^) of PvRXLR131 interacted with VvBKI1 (Fig. 5A). Thus, the 39 amino acids in the C‐terminus were sufficient for inducing the interaction.

As most functional analysis of PvRXLR131 were conducted in model plants, we further explore whether PvRXLR131 interacted with BKI1 from model plants. Homologues of *VvBKI1* in *Arabidopsis* (*AtBKI1*) and *N. benthamiana *(*NbBKI1*) were cloned and performed Y2H assays. The construct AD‐AtBKI1 or AD‐NbBKI1 was co‐transformed with BD‐PvRXLR131 into the yeast strain Y2H Gold. Similar to VvBKI1, both AtBKI1 and NbBKI1 were associated with PvRXLR131 in yeast (Fig. 5A).

### Phosphorylation of a conserved tyrosine site in BKI1 disrupts interaction with PvRXLR131

In *Arabidopsis*, AtBKI1 targets the plasma membrane and interacts with BR receptor BRI1 and ER receptor ERECTA, respectively, to inhibit BR and ER signalling. BR‐activated BRI1 phosphorylates AtBKI1 to induce its release from the plasma membrane into the cytosol, thus activating both signalling pathways (Wang *et al*., 2017). Amongst all the identified phosphorylation sites, the 211th tyrosine (Y211) has been shown to be critical for dissociation. Mutation of Y211 into phenylalanine (the non‐phosphorylizable mutant AtBKI1^Y211F^) results in constitutive plasma membrane localisation of AtBKI1 and blocks its dissociation, whereas mutation of Y211 into aspartic acid (the phospho‐mimicking mutant AtBKI1^Y211D^) was found in the cytosol (Jaillais *et al*., 2011). This tyrosine phosphorylation site was conserved in BKI1 from different plant species (Jaillais *et al*., 2011). Alignment of VvBKI1, AtBKI1 and NbBKI1 revealed that the tyrosine site was Y211, Y211 and Y199, respectively (Fig. S8, see Supporting Information). To determine whether the phosphorylation of this conserved tyrosine site affected the interaction between BKI1s and PvRXLR131, we generated the phospho‐mimicking mutants VvBKI1^Y211D^, AtBKI1^Y211D^ and NbBKI1^Y199D^ and the non‐phosphorylizable mutants VvBKI1^Y211F^, AtBKI1^Y211F^ and NbBKI1^Y199F^ (Fig. S7B, see Supporting Information) and performed Y2H assays. Notably, we found that Y211F mutants still exhibited interactions with PvRXLR131; however, Y211D mutants no longer interacted with PvRXLR131 (Fig. 5A). These data indicated that PvRXLR131 only interacted with the plasma membrane‐located BKI1s but not with the cytosol‐located BKI1s.

### PvRXLR131 suppresses BR and ER signalling *in planta*

It is reported that overexpression of AtBKI1 in *Arabidopsis* inhibits both BR and ER signalling pathways resulting in dwarf phenotype (Wang and Chory, 2006; Wang *et al*., 2017). Since *PvRXLR131*‐transgenic seedlings showed similar dwarf phenotype and PvRXLR131 was associated with AtBKI1, we speculated that PvRXLR131 interacted with AtBKI1 to suppress BR and ER signalling. Additional methods were applied to address this possibility.

**Figure 6:**
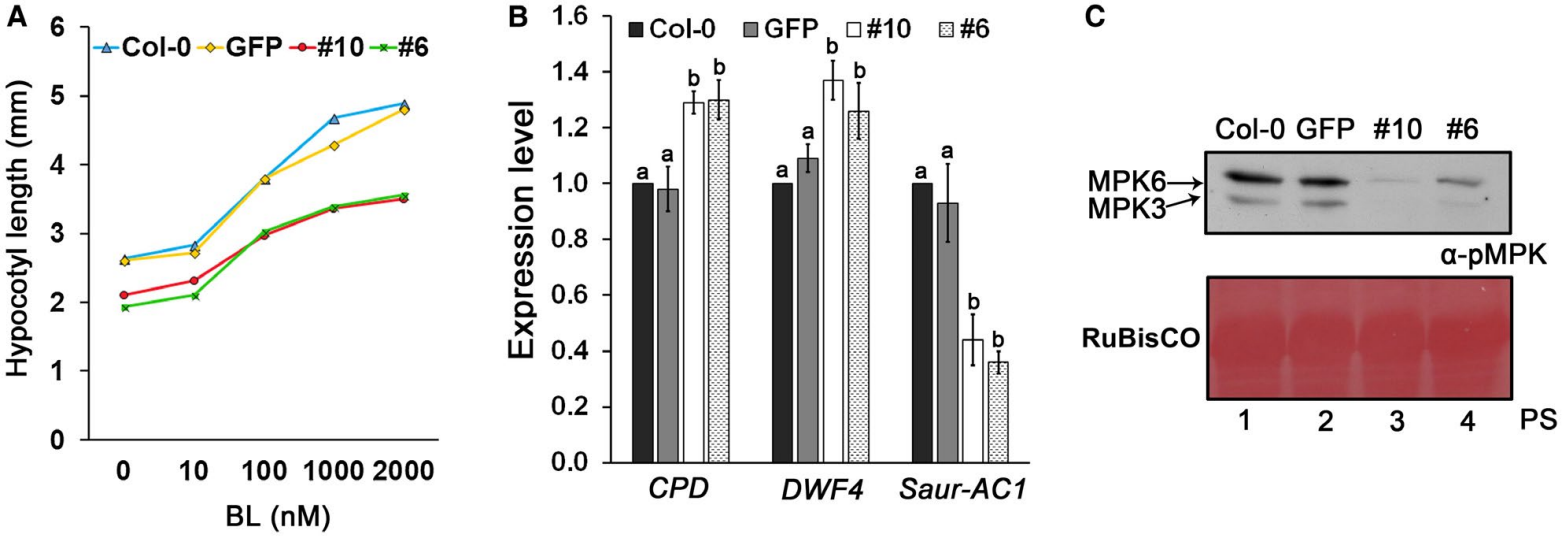
Accumulation of PvRXLR131 in *Arabidopsis* suppressed BR and ER signalling. (A) Overexpression of PvRXLR131‐GFP leads to a reduced response to BL. Hypocotyl length of at least 40 seedlings was measured. (B) Accumulation of PvRXLR131 alters the expression of BR‐responsive genes. The expression level of two BR down‐regulated genes, *CPD* and *DWF4*, was increased and that of the up‐regulated gene, *Saur‐AC1*, was reduced. Each data point represents means ± standard deviations (SDs) from three biological replicates, values with different letters show significant differences between mean values at *P* = 0.05 (LSD). *U‐box* gene (At5g15400) was used to normalize the data. (C) Comparison of MPK activity in *PvRXLR131*‐transgenic, *GFP‐*transgenic and Col‐0 *Arabidopsis*. Total proteins were extracted from 2‐week‐old seedlings; equal protein amounts were separated using SDS‐PAGE electrophoresis, and immunoblots were conducted using an anti‐p44/42‐ERK antibody. A Ponceau‐S stained RuBisCO large subunit (rbcL) served as a loading control.

First, we tested the sensitivity of *PvRXLR131*‐transgenic lines to BR. Plants were grown on medium containing different concentrations of brassinolide (BL; the most active BR), and the hypocotyl length was measured and analysed (Fig. 6A). Without BL application, *PvRXLR131*‐transgenic lines showed approximately 30% reduction in hypocotyl length, which is similar to that of *AtBKI1* transgenic seedlings (around 40% reduction, Wang *et al.*, 2006). With BL application, hypocotyl elongation was promoted in all plants. However, with increase in applied BL, the *PvRXLR131*‐transgenic lines showed reduced hypocotyl elongation compared with *GFP* and Col‐0 controls. Conversely, PvRXLR131 lines were more sensitive to brassinazole (BRZ), a BR biosynthesis inhibitor (Fig. S9, see Supporting Information). These results suggested that PvRXLR131 attenuated the sensitivity of plants to BR. Next, we determined whether overexpression of PvRXLR131 affected the transcript levels of BR‐responsive genes. As shown in Figure 6B, PvRXLR131 accumulation enhanced the expression of *CPD* and *DWF4* (two down‐regulated genes in BR signalling), but decreased the expression of *Saur*‐*AC1 *(an up‐regulated gene in BR signalling), suggesting that BR signalling pathway was suppressed by PvRXLR131. Finally, we examined the phosphorylation levels of MAPKs, which act downstream of ER signalling pathway. Both *PvRXLR131*‐transgenic lines showed lower MPK6 and MPK3 phosphorylation levels than Col‐0 and *GFP* controls (Fig. 6C), revealing that PvRXLR131 accumulation also suppressed ER signalling pathway.

In conclusion, findings from multiple studies clearly demonstrated that PvRXLR131 suppressed BR and ER signalling pathways *in planta* by interacting with BKI1.

### Virulence function of PvRXLR131 is compromised in *NbBKI1*‐silenced *N. benthamiana*

**Figure 7:**
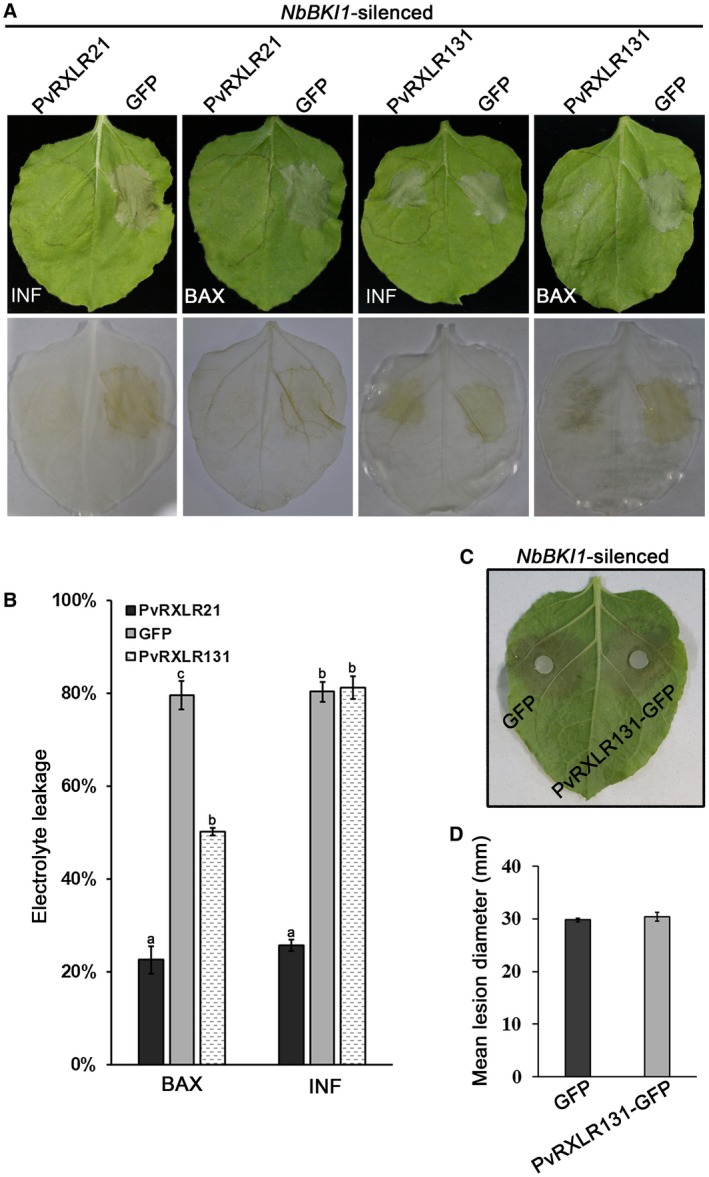
PvRXLR131 fails to suppress elicitor‐triggered cell death and promote *Phytophthora capsici* growth in *NbBKI1*‐silenced *Nicotiana benthamiana. *(A) BKI1 is required for PvRXLR131 to suppress cell death. *Agrobacterium* GV3101 carrying pGR106‐PvRXLR131, pGR106‐GFP and pGR106‐PvRXLR21 (positive control) constructs were infiltrated into *NbBKI1*‐silenced *N. benthamiana* leaves; GV3101 containing pGR106‐INF/BAX was infiltrated at the first infiltration region after 24 h. Cell death was scored 5 days after the second infiltration. The lower line indicates corresponding *N. benthamiana* leaves decolourized by alcohol blanching. Quantification of cell death by electrolyte leakage are shown (B). Each data point represents means ± standard deviations (SDs) from three biological replicates; values with different letters show significant differences between mean values at *P* = 0.01 (LSD). (C) BKI1 is required for PvRXLR131 to promote *P. capsici* growth. PvRXLR131‐GFP and GFP were transiently expressed in *NbBKI1*‐silenced *N. benthamiana* leaves; leaves were detached at 36 h, followed by inoculation of *P. capsici *discs on the undersides of leaves. Disease development was evaluated at 3 dpi and the growth area was plotted as average lesion diameter (D). Each data point represents means ± standard deviations (SDs) from three biological replicates.

To evaluate whether the virulence function of PvRXLR131 required BKI1, we conducted virus‐induced gene silencing (VIGS) of *NbBKI1* in *N. benthamiana*. Two VIGS constructs (5′ and 3′) of *NbBKI1 *were used to silence it (Fig. S10A, see Supporting Information). The results showed that expressing either of the VIGS constructs in plants could reduce the transcript level of *NbBKI1 *(Fig. S10C). *NbBKI1*‐silenced *N. benthamiana* displayed a larger size compared to the positive (TRV:*PDS*) and negative (TRV:EV) controls (Fig. S10B). We observed that in the TRV:EV control plants, PvRXLR131 could still suppress both INF‐triggered cell death (ICD) and BAX‐triggered cell death (BCD) (Fig. S11, see Supporting Information). While silencing of *NbBKI1 *completely abolished the ICD and largely attenuated BCD suppression ability of PvRXLR131, but did not alter ICD and BCD suppression ability of the positive control PvRXLR21 (Liu *et al*., 2018) (Fig. 7A). Electrolyte leakage was measured to further confirm the observed phenotypes (Fig. 7B). In addition, we found that PvRXLR131 was well expressed in both TRV:EV and *NbBKI1*‐silenced *N. benthamiana*, which eliminated the possibility that cell death was caused by the failure expression of effector protein (Fig. S12A, see Supporting Information). The failure to completely abolish the BCD suppression ability of PvRXLR131 might be because *NbBKI1* gene was not silenced completely or the BCD suppression ability of PvRXLR131 was not totally relying on NbBKI1. At the same time, we also found that silencing of *NbBKI1* led to the failure of promotion of *P. capsici* growth by PvRXLR131 (Figs 7C, D and S12B, see Supporting Information). These data indicated that the virulence function of PvRXLR131 *in planta* was directly associated with BKI1.

## Discussion

Because *P. viticola* is recalcitrant to genetic engineering, and given the inefficiency of grapevine transformation (Dubresson *et al*., 2008; Reustle and Buchholz, 2009), we used heterologous expression systems to examine the biological function of PvRXLR131. Yeast SST system was utilized to validate the secretion of PvRXLR131, because the mechanism of SP‐mediated secretion is conserved in eukaryotes, and this system has been validated in oomycetes and fungal effectors (Cheng *et al*., 2017; Liu *et al*., 2018; Oh *et al*., 2009). In addition, *C. gloeosporioides *was selected for evaluation of the role of PvRXLR131 in pathogenicity, as it is relatively simple to culture and to manipulate genetically and moreover, can produce obvious disease lesions on grapevine leaves.* PvRXLR131*‐transgenic *C. gloeosporioides* became more aggressive, demonstrating that PvRXLR131 functions well in *C. gloeosporioides*, and that this pathogen can be potentially used as a surrogate to study effectors from *P. viticola* and other biotrophic grapevine pathogens. Furthermore, the *N. benthamiana* and *Arabidopsis* model systems were used to investigate the virulence function of PvRXLR131. Because PvRXLR131 interacted with a well‐conserved plant component (BKI1), responses on non‐host species should be relevant to responses on the host.

Suppression of plant innate immunity is thought to be the primary function of most pathogen effectors, including RXLR effectors (Anderson *et al*., 2015). In some cases, ectopic expression of an effector results in an increased susceptibility to pathogens (Anderson *et al*., 2012; Li *et al*., 2016b; Turnbull *et al*., 2017). For example, Fabro *et al*. (2011) reported that ~70% of the *H. arabidopsidis* RXLR effectors could promote *P. syringae* DC3000‐LUX growth on *Arabidopsis*. In this study, we also found that transient expression of PvRXLR131 in *N. benthamiana* significantly increased* P. capsici* growth, and constitutive expression of PvRXLR131 in *Arabidopsis* promoted *P.* *syringae* DC3000 and *P.* *syringae* DC3000 (*hrcC^‐^*) growth as well as suppressed PTI‐related callose deposition. Effectors from oomycetes, especially biotrophic oomycetes, are often able to suppress defence‐related PCD in plant, which was regarded as an important mechanism for effector virulence function (Liu *et al*., 2018; Wang *et al*., 2011; Xiang *et al*., 2016). PvRXLR131 suppressed cell death triggered by BAX and INF1, highlighting the important role of this effector on virulence. Similarly, a homologue of PvRXLR131 in *P. sojae*, Avh263, can also suppress cell death elicited by BAX and INF1 (Wang *et al*., 2011). Hence, our results support the hypothesis that conserved oomycete RXLR effectors (core effectors) may share comparable modes of action in plants and play important roles in pathogen virulence (Anderson *et al*., 2012; Tomczynska *et al*., 2018).

Plant genome encodes hundreds of leucine‐rich repeat receptor‐like kinases (LRR‐RLKs) to sense environmental signals such as light, hormones and pathogens (Shiu and Bleecker, 2001). At present, AtBKI1 is the only known and well‐studied inhibitor of LRR‐RLKs (BRI1 and ERECTA) (Wang *et al*., 2017). We found that PvRXLR131 interacted with VvBKI1 and its homologues, NbBKI1 and AtBKI1. Although functional studies on VvBKI1 and NbBKI1 are lacking, it has been reported that BKI1 is functionally conserved amongst different plant species (Jiang *et al*., 2015). For example, *AtBKI1*‐transgenic *Arabidopsis* present typical dwarf phenotypes and the *Atbki1‐1* mutant is considerably larger in size. Similarly, constitutive expression of BKI1 from rice and apples yield dwarf morphologies (Jiang *et al*., 2015; Ma *et al*., 2014). Consistently, our result showed that the *NbBKI1*‐silencing *N. benthamiana* exhibited a larger size (Fig. S10).

Stably expressed PvRXLR131 in *Arabidopsis* and *N. benthamiana* resulted in dwarf plants. Pathogen effectors that alter plant phenotypes have been reported; some are toxic to plant cells, while some can modulate plant hormone levels (Block *et al*., 2010). The suppression of BR and ER signalling in *PvRXLR131*‐transgenic *Arabidopsis* indicated that PvRXLR131 targets BKI1 to suppress BR and ER hormone signalling, thereby leading to plant growth defects. In addition, PvRXLR131 significantly increased* P. capsici* growth and suppressed elicitor‐triggered cell death in *N. benthamiana*, while all these virulence function was compromised in *NbBKI1*‐silenced *N. benthamiana*. These physiological and genetic data clearly demonstrate that BKI1 is a virulence target of PvRXLR131. Many RXLR effectors have been shown to reduce disease resistance in plants, but only some of their targets have been identified and subjected to exhaustive study (Tomczynska *et al*., 2018). Our identification of BKI1 as a target enlarges the set of known operative targets of RXLR effectors and thus, further improves our understanding of the molecular mechanisms of *P. viticola* pathogenicity.

Besides growth and development regulation, ER and BR signalling are also involved in plant defence responses. The ERECTA gene induced resistance of *Arabidopsis* against bacterium and fungus (Godiard *et al*., 2003; Llorente *et al*., 2005), and the MAPK cascade (downstream of ER signalling) controls multiple defence responses, including reactive oxygen species generation, defence gene expression, and phytoalexin biosynthesis (Meng and Zhang, 2013). BR activates the expression of several immunity‐related genes and triggers defence responses against diverse pathogens in plant (Divi *et al*., 2010; Nakashita *et al*., 2003). On the other hand, BR signalling also negatively regulates disease resistance (Turnbull *et al*., 2017; De Vleeschauwer *et al*., 2012). A recent study revealed that treatment of grapevine leaves with BL significantly reduced the development of *P. viticola* sporangiophores, suggesting that grapevine BR signalling positively regulates disease resistance against *P. viticola *(Liu *et al*., 2016). Besides, perturbation of BR or ER signalling may also affect other defence‐related signalling pathways (e.g. PTI, SA, and JA/ET pathways) as there is crosstalk amongst signalling networks. Thus, the enhancement of plant susceptibility caused by PvRXLR131 may be associated with the suppression of BR and ER signalling.

Deletion assays indicated that the C‐terminus of PvRXLR131 was essential for interaction with VvBKI1, a persuasive result given that the N‐terminus of RXLR effectors is responsible for secretion and trafficking into host cells, whereas the C‐terminus is involved in function (Kamoun, 2006; Whisson *et al*., 2007). PvRXLR131 interacted solely with non‐phosphorylated BKI1s (plasma membrane‐located) and not with phosphorylated BKI1s (cytosol‐located). Furthermore, BiFC analysis also showed that PvRXLR131 interacted with VvBKI1 in the plasma membrane. It has been reported that the *Xanthomonas campestris *effector AvrAC adds uridine 5′‐monophosphate to the phosphorylation sites of BIK1 and RIPK, two receptor‐like cytoplasmic kinases, to inhibit their phosphorylation and consequently suppress downstream immune signalling (Feng *et al*., 2012). Thus, it will be interesting to determine whether PvRXLR131 inhibits the phosphorylation of BKI1 by an unidentified mechanism and prevents BKI1 dissociation.

**Figure 8:**
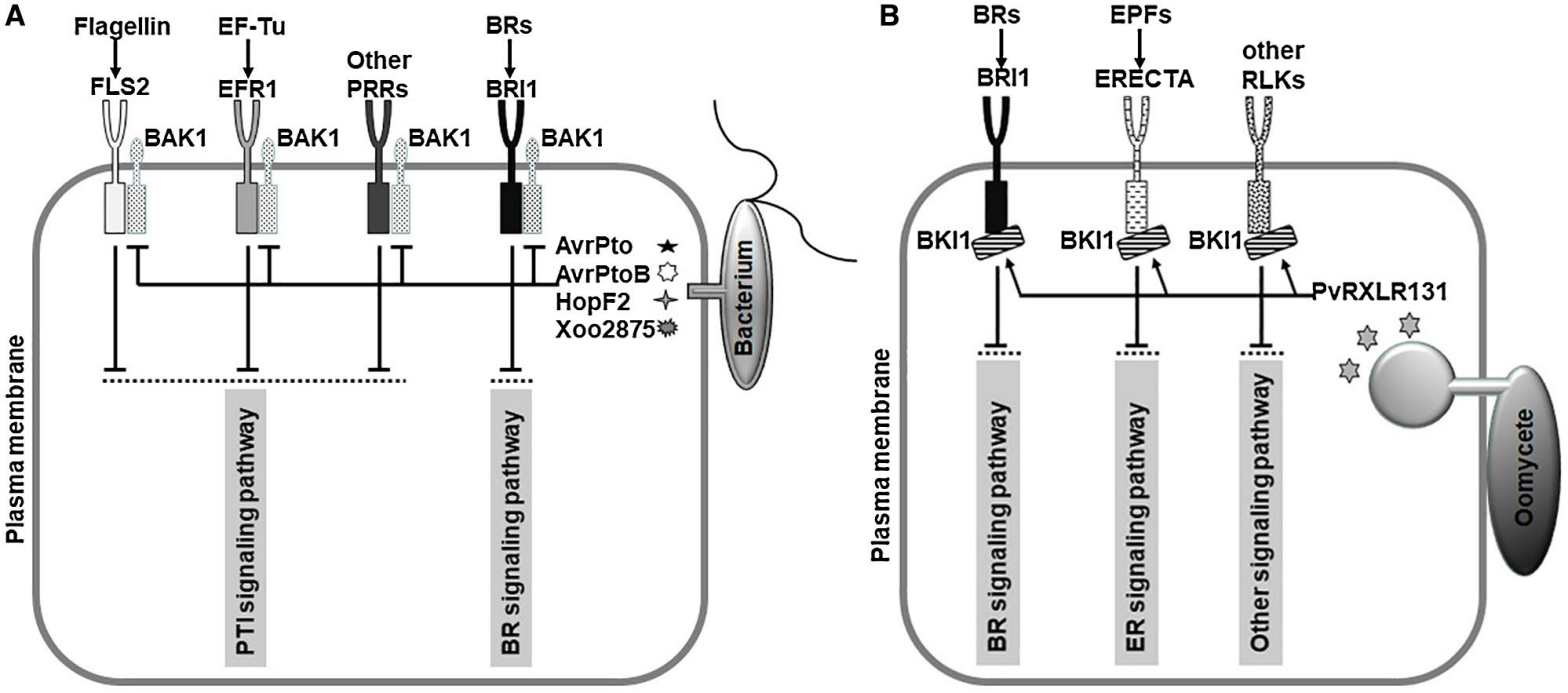
A proposed model depicting the role of bacterial effectors and oomycete effector in modulating plant defence‐related signalling. (A) The bacterial effectors AvrPto, AvrPtoB, HopF2 and Xoo2875 directly target plant BAK1, a shared signalling partner of several MAMP receptors and BR receptor BRI1, to prevent receptor complex formation and diverse downstream signalling that triggered by different MAMPs and BR. (B) The oomycete *Plasmopara viticola *effector PvRXLR131 directly target plant BKI1, a shared signalling inhibitor of BR receptor BRI1 and ER receptor ERECTA (or other RLKs), to prevent corresponding downstream signalling.

The mode of action of PvRXLR131 is somewhat similar to that of some bacterial effectors (Fig. 8). As a shared signalling partner (co‐receptor), BAK1 associates with BRI1 and PRR (FLS2 and EFR) to activate their kinase activity and enhance signalling output. The bacterial effectors AvrPto, AvrPtoB, HopF2, and Xoo2875 were evolved to target BAK1 to attenuate BR and PTI signalling (Cheng *et al*., 2011; Shan *et al*., 2008; Yamaguchi *et al*., 2013; Zhou *et al*., 2014). Much attention has been paid on decreased PTI signalling, their effect on BR has not yet been investigated, even though transgenic plants overexpressing these effectors exhibit typical BR‐insensitive phenotypes. As a shared signalling inhibitor, BKI1 directly interacts with BRI1 and ERECTA (and possibly can also interact with other untested RLKs), and inhibited their kinase activity to reduce signalling output. We have demonstrated that the *P. viticola* effector PvRXLR131 targeted BKI1 to suppress BR and ER signalling. Unlike bacterial effectors that target LRR‐RLK‐associated co‐receptor, *P. viticola* evolved effectors to target LRR‐RLK‐associated inhibitor; however, both strategies can simultaneously modulate two or more defence‐related signalling pathways.

Here, we found that *P. viticola* exploits PvRXLR131 to target LRR‐RLK‐associated inhibitor BKI1 for promoting infection. LRR‐RLKs control a large number of downstream responses and thus, interfering with LRR‐RLK functions is an effective tactic for disrupting corresponding signalling. Targeting the shared inhibitor of LRR‐RLK molecular switches by effectors to shut down/turn off two or more specific defence‐related signalling pathways to promote infection may represent a novel strategy of pathogens to conquer plant innate immunity. Our findings also support the view that hormone/peptide hormone signalling pathways are common targets of effectors from unrelated phytopathogens. In addition, because PvRXLR131 homologues exist in other oomycetes species, targeting BKI1 is probably a common strategy amongst oomycetes to avoid pathogen invasion.

## Experimental Procedures

### Plant materials and culture conditions

*Arabidopsis thaliana *ecotype Columbia (Col‐0), *N. benthamiana,* and grapevine (*Vitis vinifera* ‘Thompson Seedless’) were grown in a greenhouse at 22 °C under white light (12 h light/12 h dark).

For hypocotyl elongation assays, *Arabidopsis *seeds were spread on 1/2 Murashige and Skoog (MS) medium containing indicated concentrations of BL and BRZ (Sigma‐Aldrich, Saint Louis, MO, USA), grown in a growth chamber at 22 °C under white light (8 h light/16 h dark) and continuous darkness, respectively.

### Vector construction

*PvRXLR131* gene was cloned from the cDNA of *P. viticola*. The predicted DNA fragment encoding PvRXLR131 SP was amplified and introduced into pSUC2 vector for yeast SST assays. Full‐length *PvRXLR131* was inserted into pCB1532 for genetic transformation of *Colletotrichum gloeosporioides*. The sequence encoding mature PvRXLR131 (without SP) was introduced into the following vectors: pGR106 (for cell death suppression assays), pBI121 (for plant inoculation assays and transgenic plant production), pCAMBIA1300 (for co‐IP experiments), pXY106 (for BiFC analysis), and pColdIII (for GST pull‐down assays). Sequences encoding mature PvRXLR131 and its different truncations were inserted into pGBKT7(BD) for yeast two‐hybrid (Y2H) assays.

*VvBKI1*, *AtBKI1* and *NbBKI1* genes were cloned from cDNA of *V. vinifera*,* A. thaliana*, and *N. benthamiana*, respectively. Point mutations of *BKI1 *genes were generated by site‐directed mutagenesis using overlap extension Polymerase Chain Reaction (PCR). *BKI1* genes and their mutants were inserted into pGADT7(AD) and used in Y2H assays. *VvBKI1* was inserted into pBI121, pET30a and pXY104, for use in co‐IP experiments, GST pull‐down assays and BiFC analysis, respectively. The 5′ and 3′ DNA fragments (300 bp) of *NbBKI1* were ligated into pTRV2 for VIGS assays.

The primers used are listed in supplementary data (Tables S1 and S2). The PCR products were digested with appropriate restriction enzymes and ligated into corresponding vectors. All the recombinant plasmids were confirmed by sequencing.

### Functional validation of PvRXLR131 SP

Functional validation of PvRXLR131 SP was performed using the yeast SST system as described previously (Oh *et al*., 2009). Briefly, pSUC2‐derived plasmids were transformed into the yeast strain YTK12 by lithium acetate method (Gietz *et al*., 1995). Yeast transformants were spread on CMD‐W medium, and the positive colonies were transferred to YPRAA medium for invertase secretion assays. In 2,3,5‐triphenyltetrazolium chloride (TTC) colourimetric tests, a final concentration of 0.1% TTC was used.

### *Agrobacterium*‐mediated transient gene expression in *N. benthamiana*

The corresponding plasmids were transformed into *Agrobacterium tumefaciens* strain GV3101 by electroporation (Hellens *et al*., 2000). The transformants were grown overnight at 28 °C in Luria‐Bertani (LB) liquid medium. For pBI121, pGR106, pCAMBIA1300 and pTRV2 transformants, kanamycin (50 µg/mL) and rifampicin (50 µg/mL) were added into the medium. For pXY106 and pXY104 transformants, spectinomycin (50 µg/mL) and rifampicin (50 µg/mL) were added. Bacteria were collected by centrifugation (2500 × *g*, 5 min), washed, and resuspended in 10 mM MgCl_2_ to achieve a final optical density (OD) 600 of 0.4. For co‐expression analysis, two bacterial strains were adjusted to OD600 of 0.8 and mixed in a 1:1 ratio to achieve a final OD600 of 0.4 for each strain. *N. benthamiana* leaves were infiltrated with bacterial suspensions using syringes without needles.

### Generation of transgenic *C. gloeosporioides* and plants

Genetic transformation of *C. gloeosporioides* was performed according to the method described by Chung *et al*. (2002). Protoplasts were prepared from young hyphae using cell wall‐degrading enzymes. pCB1532‐PvRXLR131 and pCB1532‐GFP plasmids were transformed into prepared protoplasts, and the transformants were then plated on regeneration agar medium with 10 µg/mL sulfonylurea. Colonies that appeared after 4 days–6 days were transferred to potato dextrose agar (PDA) medium containing 50 µg/mL sulfonylurea, and then cultured under non‐selective conditions (without sulfonylurea) for 5 weeks (transferred to fresh medium once a week). Isolates that re‐grew on sulfonylurea‐containing PDA were preserved. Gene expression was confirmed by Reverse Transcription (RT)‐PCR and fluorescence microscopy.

*A. thaliana* and *N. benthamiana* transgenic plants were generated through methods described by Clough and Bent (1998) and Horsch (1985), respectively. *Agrobacterium *containing pBI121‐PvRXLR131‐GFP was used for stable transformation.

### Plant inoculation assays

*P. viticola* infection was performed as described previously (Xiang *et al*., 2016). The susceptible grapevine (*Vitis vinifera* ‘Thompson Seedless’) leaf discs were inoculated with *P. viticola* ‘JL‐7‐2’ (30 µL of spore suspension with a concentration of 10^5^ spores/mL). Samples were harvested at 0, 6, 12, 24, 36, 48, 60, 72, 96 and 120 hpi, and used for RNA extraction.

*C. gloeosporioides* and *P. capsici* were routinely cultured on PDA and oatmeal agar (OA) plates, respectively, for 7 days at 25 °C. Mycelial discs with a diameter of 7 mm were prepared and inoculated onto the abaxial side of detached leaves. The inoculated leaves were kept in a moist chamber at 25 °C. Necrotic lesions were monitored after inoculation.

*P. syringae* pathovar tomato DC3000 and its mutant strain DC3000 (*hrcC^−^*) were cultured in King's B (KB) broth with rifampicin (50 µg/mL). Bacteria were collected by centrifugation (2500 × *g*, 5 min), washed, and resuspended in 10 mM MgCl_2_ to achieve a concentration of 10^6^ colony forming unit (CFU)/mL^−1^. *Arabidopsis* leaves (5‐week‐old) were syringe‐infiltrated with bacterial suspensions. Leaf bacterial number was determined at 1 h (0 days post‐inoculation; 0 dpi) and 48 h (2 dpi) after inoculation. The plant tissues were ground in distilled water, and serial 10‐fold dilutions were spread on KB plates and kept at 28 °C for 2 days. The number of generated colonies was counted.

### Yeast two‐hybrid assay

A Gal4‐based Y2H screening was performed as described below. *P. viticola*‐infected grapevine cDNA library was constructed in pGADT7 vector (Shanghai Oebiotech, Shanghai, China) expressing prey protein in frame with activation domain (AD). pGBKT7‐PvRXLR131, which encodes mature PvRXLR131 fused with binding domain (BD), was used as a bait to screen the cDNA library. Preparation and transformation of competent yeast cells of Y2H Gold strain were carried out using Yeastmaker Yeast Transformation system 2 kit (Clontech, Mountain View, CA, USA). Potential yeast transformants containing cDNA clones interacting with PvRXLR131 were selected using the quadruple dropout medium (QDO; SD/‐Trp/‐Leu/‐His/‐Ade).

To verify the interaction between two proteins, both prey plasmid and bait plasmid were co‐transformed into the yeast strain Y2H Gold. Yeast transformants were cultured on double dropout medium (DDO; SD/‐Leu/‐Trp), and the positive colonies were transferred to QDO medium supplemented with X‐α‐Gal and Aureobasidin A (QDO/X/A). The colonies grew and turned blue on QDO/X/A plates containing two interacting proteins.

### Protein extraction and Western blot analysis

The plant tissues were harvested, frozen in liquid nitrogen and ground into powder. Proteins were extracted from the powder using lysis buffer (50 mM Tris‐HCl pH 7.5, 150 mM NaCl, 1 mM EDTA, 0.5% NP‐40, 1 × protease inhibitor cocktail, 5 µM MG132) and quantified by Bradford reagent. Equal amounts of protein were separated by SDS‐PAGE, and then transferred to nitrocellulose membrane. The membrane was blocked with 5% non‐fat milk in TBST, and then incubated with the appropriate primary antibody for 3 h, followed by incubation with secondary antibody for 1 h. The primary antibodies included anti‐GFP, anti‐His, anti‐GST (TransGen Biotech, Beijing, China), anti‐Flag (Sigma‐Aldrich), and anti‐p44/42‐ERK (Cell Signaling Technology, Danvers, MA, USA). The secondary antibodies were anti‐mouse IgG and anti‐rabbit IgG (Sigma‐Aldrich). The signal was detected using a Pierce ECL Western Blotting Substrate (Thermo Scientific, Rockford, IL, USA).

### Co‐immunoprecipitation

VvBKI1‐GFP was transiently expressed alone or co‐expressed with PvRXLR131‐Flag in *N. benthamiana*. The samples were harvested after 2 days, and total proteins were extracted. Equal amounts of total proteins were incubated with pre‐washed anti‐Flag magnetic beads for 1.5 h on a rotator at 4 °C. The beads were separated using a magnetic frame and washed six times with TBS containing 0.5% NP‐40. Immunoprecipitates were analysed by Western blotting.

### GST pull‐down assay

*Escherichia coli* Rosetta (DE3) was used for protein prokaryotic expression. GST‐ and His‐tagged proteins were purified by Glutathione MagBeads and Ni‐Charged MagBeads (GenScript, Nanjing, China), respectively. Purified His‐tagged protein (10 µg) and/or GST‐tagged protein (10 µg) were mixed and incubated with 30 µL Glutathione MagBeads in phosphate‐buffered saline (PBS) for 3 h. MagBeads were separated using a magnetic frame and washed five times with 1 × PBS containing 0.5% NP‐40. The bound proteins were analysed by Western blotting.

### Confocal microscopy

Verification of *GFP*‐transgenic *C. gloeosporioides* and BiFC analysis were performed by confocal microscopy. Hyphae or patches of leaves were mounted in distilled water on slides, and analysed using a Leica TCS SP8 confocal microscope. GFP and YFP were imaged at excitation/emission wavelengths of 488/500 nm–530 nm and 514/530 nm–575 nm, respectively.

### VIGS assay

Two largest leaves of *N. benthamiana* (3‐week‐old) were co‐infiltrated with *Agrobacterium* strains containing pTRV1 vector and pTRV2, pTRV2‐5′*NbBKI1*, pTRV2‐3′*NbBKI1* or pTRV2‐*PDS*. The agroinfiltrated plants were then grown for 3 weeks before using for *P. capsici* infection and cell death assays. Gene silencing was confirmed by RT‐PCR.

### Electrolyte leakage assay

Cell death of *N. benthamiana* was quantified by electrolyte leakage and performed as described in our previous study (Xiang *et al*., 2017).

### Callose deposition assay

*Arabidopsis *leaves (5‐week‐old) were infiltrated with 1 μM flg22. The samples were harvested at 12 h and stained with aniline blue for callose (Adam and Somerville, 1996). The stained callose was observed and imaged under a Leica DM2500 fluorescence microscope. The number of callose deposition was determined as described previously (Li *et al*., 2016b).

### Quantitative RT‐PCR analysis

Total RNA was extracted using a plant RNA kit (Omega, Norcross, GA, USA), and first‐strand cDNA was synthesized using One‐Step gDNA Removal and cDNA Synthesis SuperMix kit (TransGen Biotech). cDNA was combined with SYBR Green Fast qPCR Mix (Bio‐rad, Hercules, CA, USA), and the primers for each gene were added (Table S3). Quantitative RT‐PCR was performed on a Thermal cycler CFX96 real‐time machine (Bio‐Rad) and in triplicate.

## Supporting information


**Fig. S1** Sequence alignment of PvRXLR131 and its homologues in oomycetes. The alignment was conducted by using ClustalW in MEGA7 software. Conserved sites at 60% level are shown in background colour. The predicted signal peptide and RXLR‐dEER motif are indicated. Species names are marked in the round brackets and the corresponding accession numbers are marked on the left.

**Fig. S2** Detection of the *PvRXLR131* gene. (A) *PvRXLR131* is expressed during infection. The leaf discs of the susceptible *Vitis vinifera* ‘Thompson Seedless’ were drop‐inoculated with the spore suspension of *Plasmopara viticola* and harvested at indicated time points post‐inoculation. The *PvRXLR131* transcript levels in the infected leaf discs were quantified by quantitative Polymerase Chain Reaction (qPCR). The transcripts of *PvActin* (*P. viticola* actin) were used as the reference. The growth of *P. viticola* was monitored and plotted as the relative quantity of *PvActin* to *VvActin* (*V. vinifera* actin) (B). The error bars represent means ± standard deviations (SDs) from three replicates. (C) PvRXLR131 is detected in the gDNA samples from different *P. viticola* isolates. Each band (477 bp) indicates a PCR product from the corresponding *P. viticola* isolate gDNA. The PCR products were confirmed by sequencing. The primers of full‐length effector genes were used. M, DNA marker.

**Fig. S3** Characterization of* Colletotrichum gloeosporioides* transformants by fluorescence microscopy and Reverse Transcription‐Polymerase Chain Reaction (RT‐PCR). (A) Green fluorescence is detected in *GFP*‐transgenic *C. gloeosporioides. *Fungal hyphae were used for analysis. Bars = 20µm. (B) RT‐PCR expression analysis of *C. gloeosporioides *transformants. One line with high expression level of *GFP*, and two lines with high expression level of *PvRXLR131 *(#3, #4*) *are shown. *Actin* was used as an endogenous reference gene.

**Fig. S4** *PvRXLR131*‐transgenic *Arabidopsis* display dwarf phenotype. A 5‐week‐old and 7‐week‐old *Arabidopsis* (*PvRXLR131*‐transgenic, *GFP*‐transgenic and Col‐0) were photographed.

**Fig. S5** PvRXLR131 affects callose deposition.* PvRXLR131*‐transgenic* Arabidopsis* and Col‐0 were treated with flg22.* PvRXLR131*‐transgenic *Arabidopsis* show reduced callose deposition compared with Col‐0 after flg22 treatment (A and B). Each data represents means ± standard deviations (SDs) from three replicates; asterisks indicate signiﬁcant differences from Col‐0 (**P < 0.01. Student's *t*‐test).

**Fig. S6** Detection of protein expression. (A) Immunoblot analysis of proteins from *Nicotiana benthamiana* leaves transiently expressing GFP and PvRXLR131‐Flag from the pGR106 vector. (B) Immunoblot analysis of proteins from *N. benthamiana* leaves transiently expressing GFP and PvRXLR131‐GFP from the pBI121 vector. Ponceau‐S (PS) stained RuBisCO large subunit (rbcL) serves as a loading control.

**Fig. S7** Schematic diagrams of deletion mutants for PvRXLR131 and point mutants for diverse BKI1s. (A) and point mutants for BKI1s (B). PvRXLR131(f), full length of PvRXLR131. PvRXLR131(m), mature PvRXLR131. The numbers represent amino acid position counting from the N‐terminus.

**Fig. S8** Alignment of amino acid sequences of VvBKI1, NbBKI1 and AtBKI1. The alignment was conducted by using ClustalW in MEGA7 software. The conserved sites at 60% level are showed in background colour. Conserved tyrosine site is labelled in red box.

**Fig. S9** Overexpression of PvRXLR131‐GFP leads to an enhanced response to BRZ. Hypocotyl length of at least 40 seedlings were measured. Scale bar = 10 mm.

**Fig. S10** Silencing of *NbBKI1* in *Nicotiana benthamiana. *(A) Virus‐induced gene silencing (VIGS) constructs of *NbBKI1. *~300 bp fragment of 5′‐ and 3′‐*NbBKI1 *were introduced into pTRV2 vector and used for *NbBKI1* silencing. (B)* NbBKI1*‐silenced plants showed significantly larger size compared with TRV:EV and TRV:*PDS* controls. (C) *NbBKI1* silencing was detected by semi‐quantitative Reverse Transcription‐Polymerase Chain Reaction (RT‐PCR). Both of the 5′‐ and 3′‐*NbBKI1 *silencing constructs resulted in reduction of expression level of *NbBKI1 *gene. *GAPDH* gene was used as an endogenous reference gene.

**Fig. S11** PvRXLR131 suppresses elicitor‐triggered cell death in TRV:EV leaves. (A) *Agrobacterium* GV3101 carrying pGR106‐PvRXLR131, pGR106‐GFP and pGR106‐PvRXLR21 (positive control) constructs were infiltrated into TRV:EV *Nicotiana benthamiana* leaves; GV3101 containing pGR106‐INF/BAX was infiltrated at the first infiltration region after 24_h. Cell death was scored 5_days after the second infiltration. The lower line indicates corresponding decolourized *N. benthamiana* leaves. Quanti?cation of cell death by electrolyte leakage are shown (B). Each data point represents means ± standard deviations (SDs) from three biological replicates; values with different letters show significant differences between mean values at *P* = 0.01 (LSD).

**Fig. S12** Detection of protein expression. (A) Immunoblot analysis of proteins from TRV:EV (EV) and *NbBKI1*‐silenced (BKI1si) *Nicotiana benthamiana* transiently expressing PvRXLR131‐Flag, PvRXLR21‐Flag and GFP from the pGR106 vector. (B) Immunoblot analysis of proteins from *N. benthamiana* leaves transiently expressing GFP and PvRXLR131‐GFP from the pBI121 vector. Ponceau‐S (PS) stained RuBisCO large subunit (rbcL) serves as a loading control.

**Table S1** Primers for PvRXLR131 plasmid construction.

**Table S2** Primers for BKI1 plasmid construction.

**Table S3** Primers for quantitative Polymerase Chain Reaction (qPCR).

**Table S4** Other primers used.
