# Supplementary material for: Plasmopara viticola effector PvRXLR131 suppresses plant immunity by targeting plant receptor‐like kinase inhibitor BKI1
Source: Mol Plant Pathol. 2019 Apr 4;20(6):765–83. doi: 10.1111/mpp.12790 (PMC6637860; doi:10.1111/mpp.12790)
Supplement: Supplementary file 1 — Fig. S1 Sequence alignment of PvRXLR131 and its homologues in oomycetes. The alignment was conducted by using ClustalW in MEGA7 software. Conserved sites at 60% level are shown in background colour. The predicted signal peptide and RXLR‐dEER motif are indicated. Species names are marked in the round brackets and the corresponding accession numbers are marked on the left. [file MPP-20-765-s001.pdf]

FIGURE S1

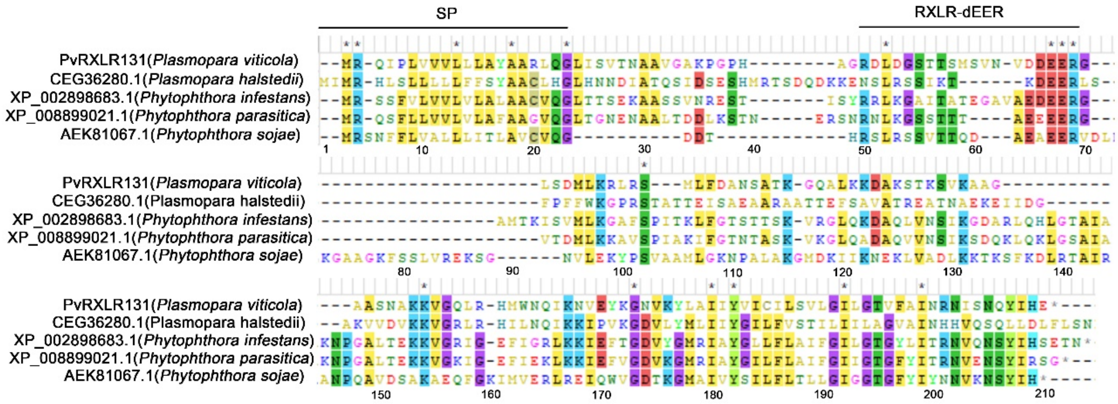

S1 Fig. Sequence alignment of PvRXLR131 and its homologs in oomycetes. The alignment was conducted by using ClustalW in MEGA7 software. Conserved sites at 60% level are shown in background color. The predicted signal peptide and RXLR-dEER motif are indicated. Species names are marked in the round brackets and the corresponding accession numbers are marked on the left.
