# Supplementary material for: Plasmopara viticola effector PvRXLR131 suppresses plant immunity by targeting plant receptor‐like kinase inhibitor BKI1
Source: Mol Plant Pathol. 2019 Apr 4;20(6):765–83. doi: 10.1111/mpp.12790 (PMC6637860; doi:10.1111/mpp.12790)
Supplement: Supplementary file 2 — Fig. S2 Detection of the PvRXLR131 gene. (A) PvRXLR131 is expressed during infection. The leaf discs of the susceptible Vitis vinifera ‘Thompson Seedless’ were drop‐inoculated with the spore suspension of Plasmopara viticola and harvested at indicated time points post‐inoculation. The PvRXLR131 transcript levels in the infected leaf discs were quantified by quantitative Polymerase Chain Reaction (qPCR). The transcripts of PvActin (P. viticola actin) were used as the reference. The growth of P. viticola was monitored and plotted as the relative quantity of PvActin to VvActin (V. vinifera actin) (B). The error bars represent means ± standard deviations (SDs) from three replicates. (C) PvRXLR131 is detected in the gDNA samples from different P. viticola isolates. Each band (477 bp) indicates a PCR product from the corresponding P. viticola isolate gDNA. The PCR products were confirmed by sequencing. The primers of full‐length effector genes were used. M, DNA marker. [file MPP-20-765-s002.pdf]

FIGURE S2

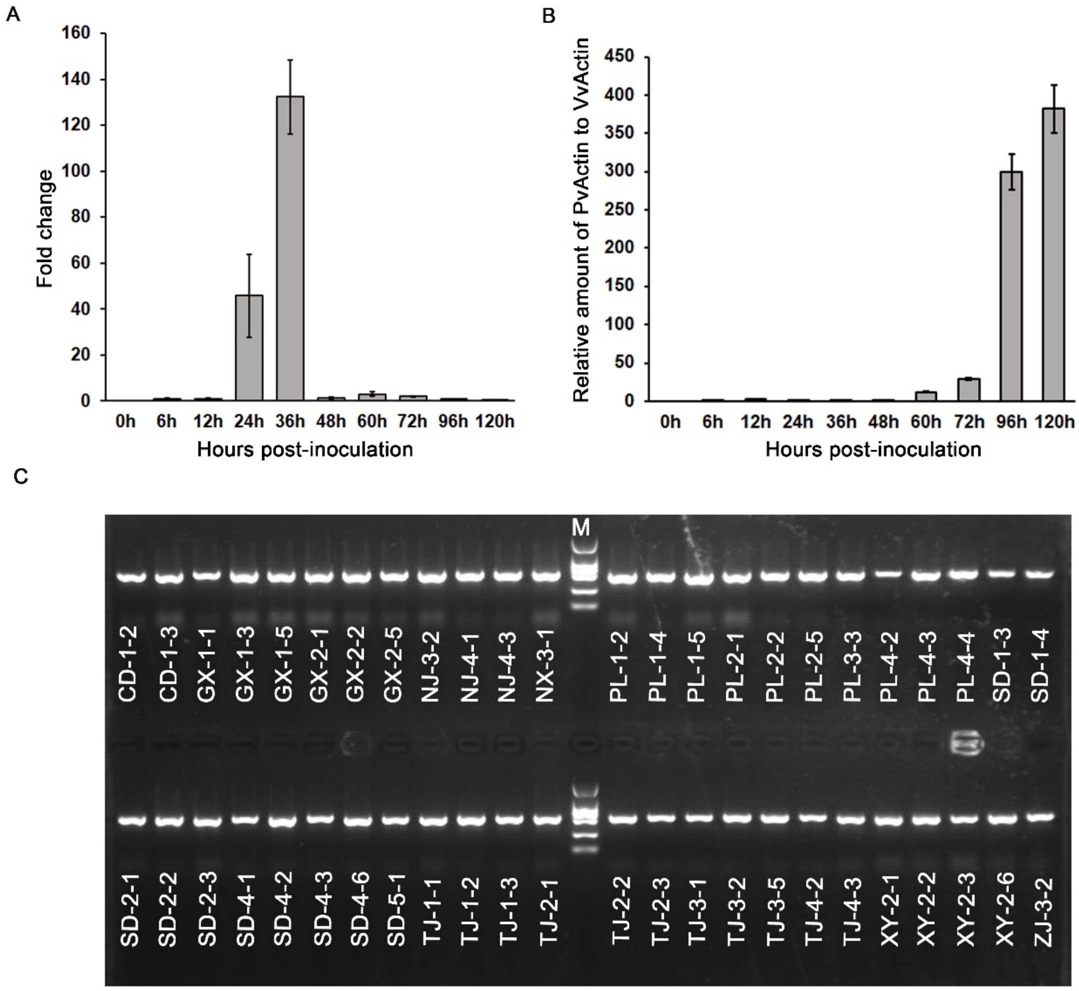

S2 Fig. Detection of the *PvRXLR131* gene. (A) *PvRXLR131* is expressed during infection. The leaf discs of the susceptible *Vitis vinifera* 'Thompson Seedless' were drop-inoculated with the spore suspension of *Plasmopara viticola* and harvested at indicated time-points post inoculation.The *PvRXLR131* transcript levels in the infected leaf discs were quantified by qPCR. The transcripts of *PvActin* (*P. viticola* actin) were used as the reference. The growth of *P. viticola* was monitored and plotted as the relative quantity of *PvActin* to *VvActin* (*V. vinifera* actin) (B). The error bars represent means ± SD from three replicates. (C) *PvRXLR131* is detected in the gDNA samples from different *P. viticola* isolates. Each band (477 bp) indicates a PCR product from the corresponding *P. viticola* isolate gDNA. The PCR products were confirmed by sequencing. The primers of full-length effector genes were used. M, DNA marker.
