# Supplementary material for: Plasmopara viticola effector PvRXLR131 suppresses plant immunity by targeting plant receptor‐like kinase inhibitor BKI1
Source: Mol Plant Pathol. 2019 Apr 4;20(6):765–83. doi: 10.1111/mpp.12790 (PMC6637860; doi:10.1111/mpp.12790)
Supplement: Supplementary file 3 — Fig. S3 Characterization of Colletotrichum gloeosporioides transformants by fluorescence microscopy and Reverse Transcription‐Polymerase Chain Reaction (RT‐PCR). (A) Green fluorescence is detected in GFP‐transgenic C. gloeosporioides. Fungal hyphae were used for analysis. Bars = 20µm. (B) RT‐PCR expression analysis of C. gloeosporioides transformants. One line with high expression level of GFP, and two lines with high expression level of PvRXLR131 (#3, #4) are shown. Actin was used as an endogenous reference gene. [file MPP-20-765-s003.pdf]

**FIGURE S3**

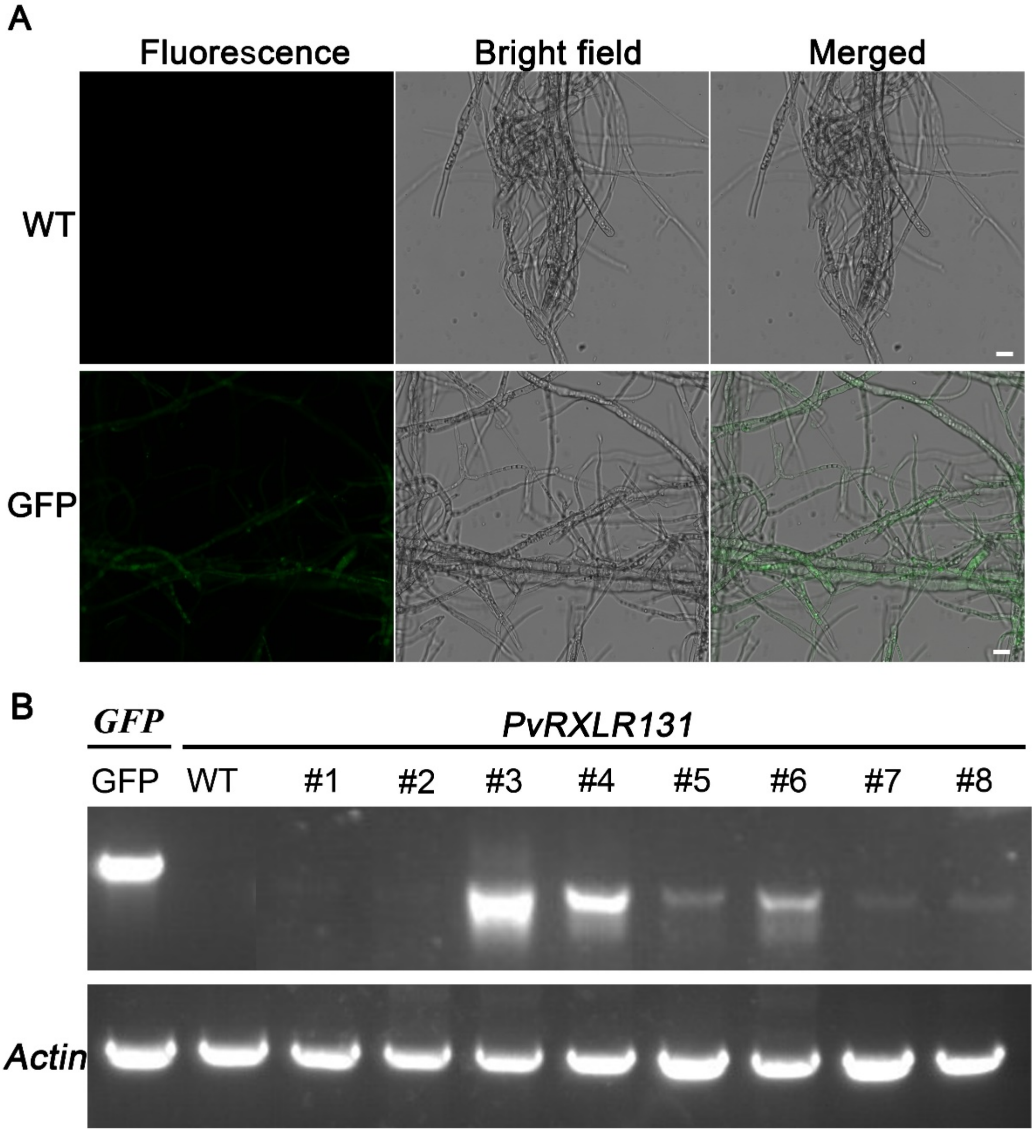


**S3 Fig.** Characterization of *Colletotrichum gloeosporioides* transformants by fluorescence microscopy and RT-PCR. (A) Green fluorescence is detected in *GFP*-transgenic *C. gloeosporioides.* Fungal hyphae were used for analysis. Bars=20μm. (B) RT-PCR expression analysis of *C. gloeosporioides* transformants. One line with high expression level of *GFP*, and two lines with high expression level of *PvRXLR131* (#3, #4*)* are shown. *Actin* was used as an endogenous reference gene.
