# Supplementary material for: Plasmopara viticola effector PvRXLR131 suppresses plant immunity by targeting plant receptor‐like kinase inhibitor BKI1
Source: Mol Plant Pathol. 2019 Apr 4;20(6):765–83. doi: 10.1111/mpp.12790 (PMC6637860; doi:10.1111/mpp.12790)
Supplement: Supplementary file 4 — Fig. S4 PvRXLR131‐transgenic Arabidopsis display dwarf phenotype. A 5‐week‐old and 7‐week‐old Arabidopsis (PvRXLR131‐transgenic, GFP‐transgenic and Col‐0) were photographed. [file MPP-20-765-s004.pdf]

FIGURE S4

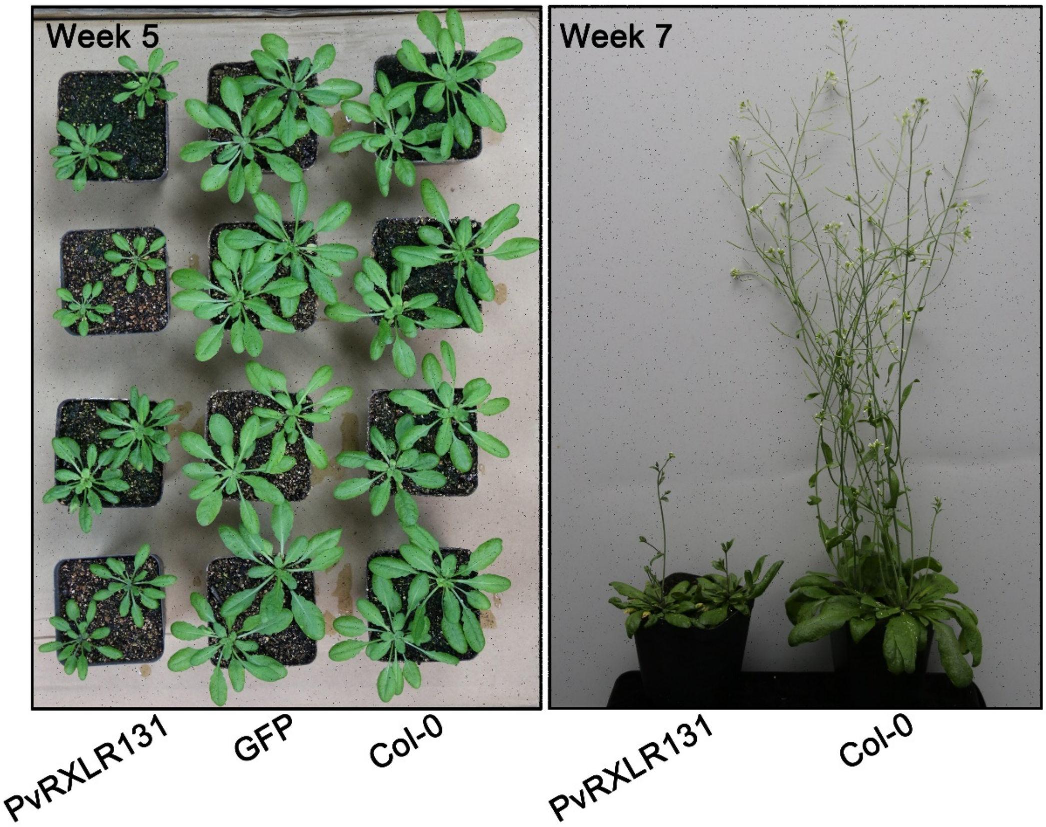

S4 Fig. *PvRXLR131*-transgenic *Arabidopsis* display dwarf phenotype. Five-week-old and Seven-week-old *Arabidopsis* (*PvRXLR131*-transgenic, *GFP*-transgenic and Col-0) were photographed.
