# Supplementary material for: Plasmopara viticola effector PvRXLR131 suppresses plant immunity by targeting plant receptor‐like kinase inhibitor BKI1
Source: Mol Plant Pathol. 2019 Apr 4;20(6):765–83. doi: 10.1111/mpp.12790 (PMC6637860; doi:10.1111/mpp.12790)
Supplement: Supplementary file 5 — Fig. S5 PvRXLR131 affects callose deposition. PvRXLR131‐transgenic Arabidopsis and Col‐0 were treated with flg22. PvRXLR131‐transgenic Arabidopsis show reduced callose deposition compared with Col‐0 after flg22 treatment (A and B). Each data represents means ± standard deviations (SDs) from three replicates; asterisks indicate signiﬁcant differences from Col‐0 (**P < 0.01. Student's t‐test). [file MPP-20-765-s005.pdf]

FIGURE S5

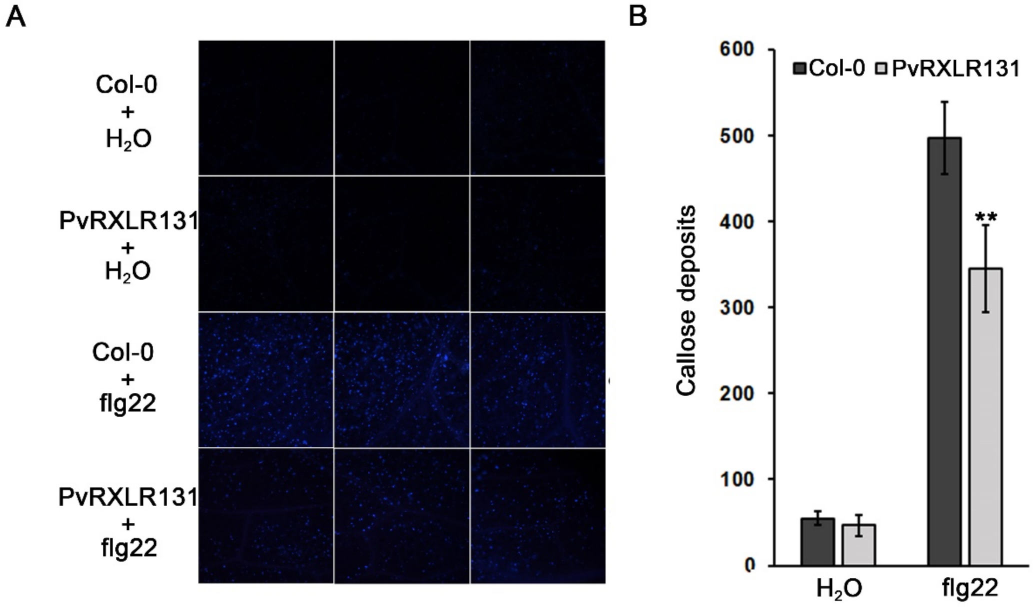

S5 Fig. PvRXLR131 affects callose deposition in *Arabidopsis*. *PvRXLR131*-transgenic *Arabidopsis* and Col-0 were treated with flg22. *PvRXLR131*-transgenic *Arabidopsis* show reduced callose deposition compared with Col-0 after flg22 treatment (A, B). Each data represents means ± SD from three replicates; asterisks indicate significant differences from Col-0 (**$P < 0.01$. Student's t test).
