# Supplementary material for: Plasmopara viticola effector PvRXLR131 suppresses plant immunity by targeting plant receptor‐like kinase inhibitor BKI1
Source: Mol Plant Pathol. 2019 Apr 4;20(6):765–83. doi: 10.1111/mpp.12790 (PMC6637860; doi:10.1111/mpp.12790)
Supplement: Supplementary file 6 — Fig. S6 Detection of protein expression. (A) Immunoblot analysis of proteins from Nicotiana benthamiana leaves transiently expressing GFP and PvRXLR131‐Flag from the pGR106 vector. (B) Immunoblot analysis of proteins from N. benthamiana leaves transiently expressing GFP and PvRXLR131‐GFP from the pBI121 vector. Ponceau‐S (PS) stained RuBisCO large subunit (rbcL) serves as a loading control. [file MPP-20-765-s006.pdf]

FIGURE S6

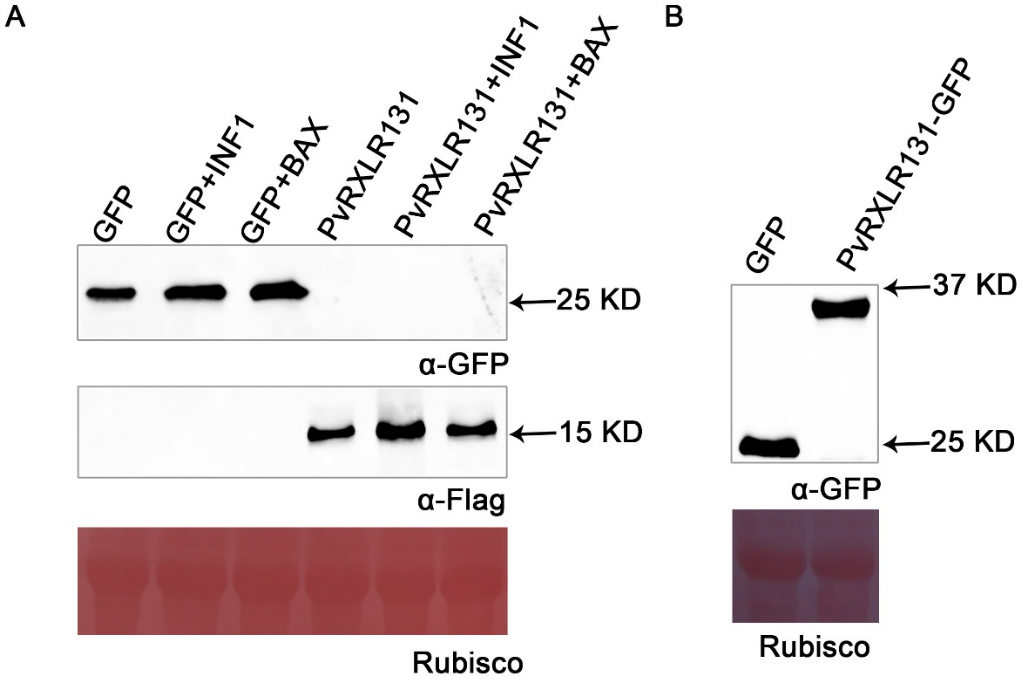

S6 Fig. Detection of protein expression. (A) Immunoblot analysis of proteins from *Nicotiana benthamiana* leaves transiently expressing GFP and PvRXLR131-Flag from the pGR106 vector. (B) Immunoblot analysis of proteins from *N. benthamiana* leaves transiently expressing GFP and PvRXLR131-GFP from the pBI121 vector. Ponceau-S (PS) stained rubisco large subunit serves as a loading control.
