# Supplementary material for: Plasmopara viticola effector PvRXLR131 suppresses plant immunity by targeting plant receptor‐like kinase inhibitor BKI1
Source: Mol Plant Pathol. 2019 Apr 4;20(6):765–83. doi: 10.1111/mpp.12790 (PMC6637860; doi:10.1111/mpp.12790)
Supplement: Supplementary file 7 — Fig. S7 Schematic diagrams of deletion mutants for PvRXLR131 and point mutants for diverse BKI1s. (A) and point mutants for BKI1s (B). PvRXLR131(f), full length of PvRXLR131. PvRXLR131(m), mature PvRXLR131. The numbers represent amino acid position counting from the N‐terminus. [file MPP-20-765-s007.pdf]

FIGURE S7

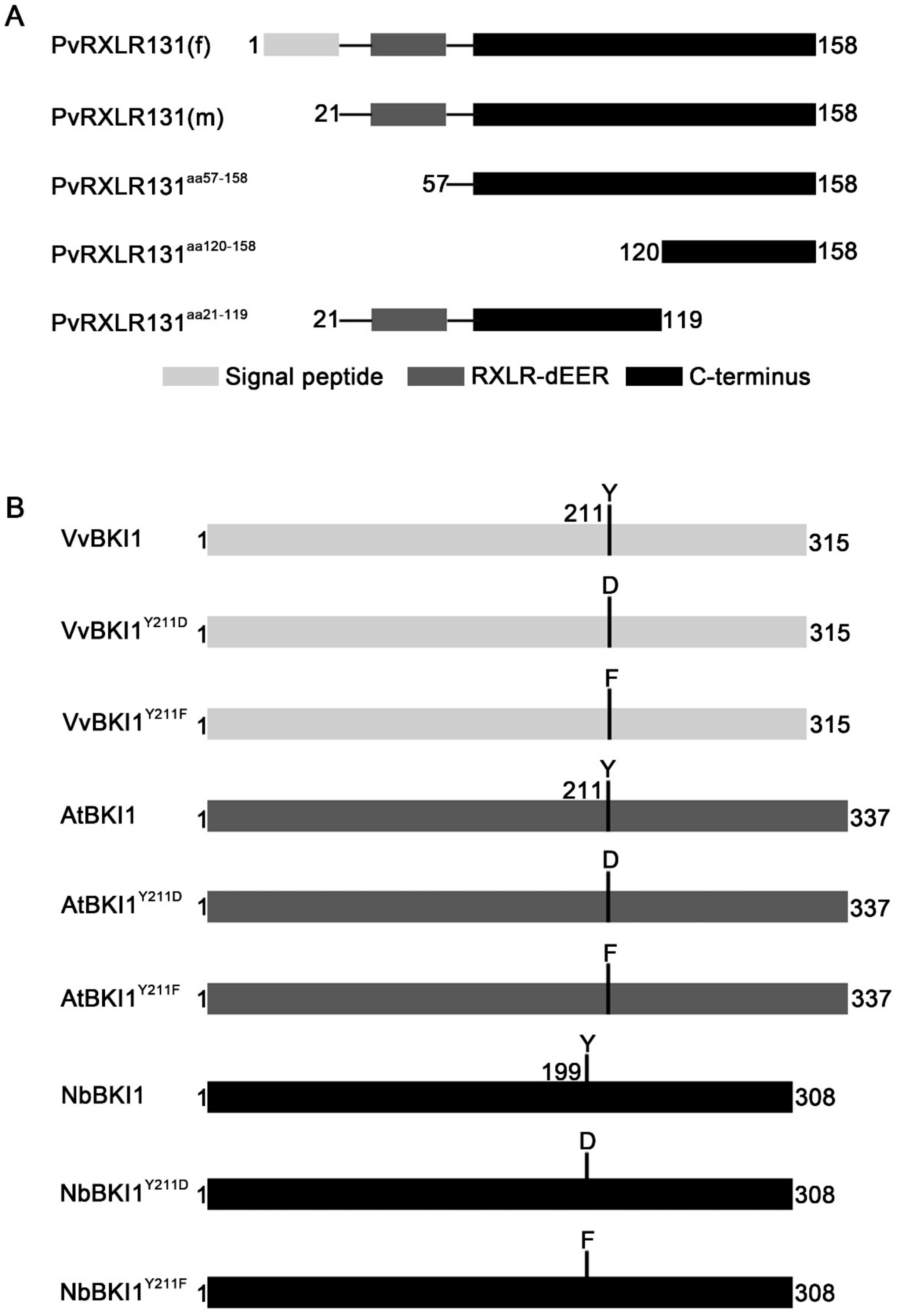

S7 Fig. Schematic diagrams of deletion mutants for PvRXLR131 (A) and point mutants for BKI1s (B). PvRXLR131(f), full length of PvRXLR131. PvRXLR131(m), mature PvRXLR131. The numbers represent amino acid position counting from the N terminus.
