# Supplementary material for: Plasmopara viticola effector PvRXLR131 suppresses plant immunity by targeting plant receptor‐like kinase inhibitor BKI1
Source: Mol Plant Pathol. 2019 Apr 4;20(6):765–83. doi: 10.1111/mpp.12790 (PMC6637860; doi:10.1111/mpp.12790)
Supplement: Supplementary file 8 — Fig. S8 Alignment of amino acid sequences of VvBKI1, NbBKI1 and AtBKI1. The alignment was conducted by using ClustalW in MEGA7 software. The conserved sites at 60% level are showed in background colour. Conserved tyrosine site is labelled in red box. [file MPP-20-765-s008.pdf]

FIGURE S8

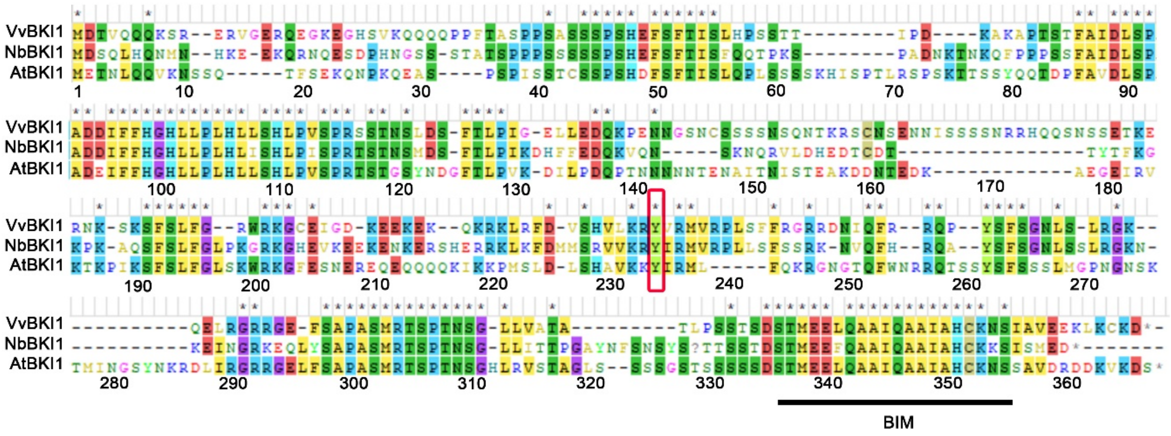

S8 Fig. Alignment of amino acid sequences of VvBKI1, NbBKI1, and AtBKI1.The alignment was conducted by using ClustalW in MEGA7 software. The conserved sites at 60% level are showed in background color. Conserved tyrosine site is labeled in red box.
