# Supplementary material for: Plasmopara viticola effector PvRXLR131 suppresses plant immunity by targeting plant receptor‐like kinase inhibitor BKI1
Source: Mol Plant Pathol. 2019 Apr 4;20(6):765–83. doi: 10.1111/mpp.12790 (PMC6637860; doi:10.1111/mpp.12790)
Supplement: Supplementary file 9 — Fig. S9 Overexpression of PvRXLR131‐GFP leads to an enhanced response to BRZ. Hypocotyl length of at least 40 seedlings were measured. Scale bar = 10 mm. [file MPP-20-765-s009.pdf]

FIGURE S9

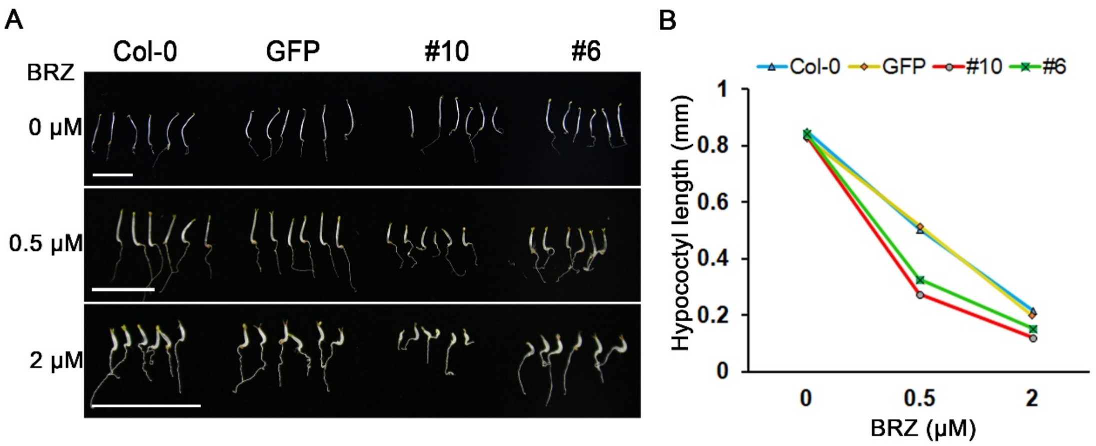

S9 Fig. Overexpression of PvRXLR131-GFP leads to an enhanced response to BRZ. Hypocotyl length of at least 40 seedlings were measured. Scale bar = 10 mm.
