# Supplementary material for: Plasmopara viticola effector PvRXLR131 suppresses plant immunity by targeting plant receptor‐like kinase inhibitor BKI1
Source: Mol Plant Pathol. 2019 Apr 4;20(6):765–83. doi: 10.1111/mpp.12790 (PMC6637860; doi:10.1111/mpp.12790)
Supplement: Supplementary file 10 — Fig. S10 Silencing of NbBKI1 in Nicotiana benthamiana. (A) Virus‐induced gene silencing (VIGS) constructs of NbBKI1. ~300 bp fragment of 5′‐ and 3′‐NbBKI1 were introduced into pTRV2 vector and used for NbBKI1 silencing. (B) NbBKI1‐silenced plants showed significantly larger size compared with TRV:EV and TRV:PDS controls. (C) NbBKI1 silencing was detected by semi‐quantitative Reverse Transcription‐Polymerase Chain Reaction (RT‐PCR). Both of the 5′‐ and 3′‐NbBKI1 silencing constructs resulted in reduction of expression level of NbBKI1 gene. GAPDH gene was used as an endogenous reference gene. [file MPP-20-765-s010.pdf]

FIGURE S10

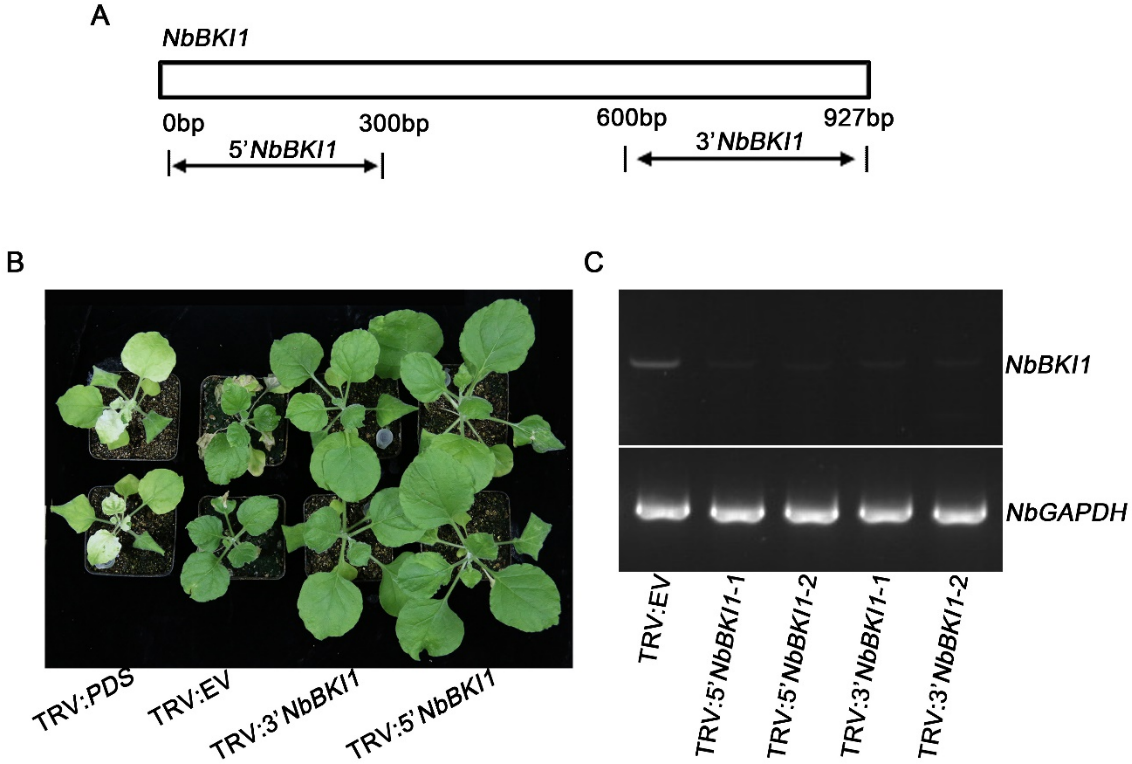

S10 Fig. Silencing of *NbBKI1* in *Nicotiana benthamiana.* (A) VIGS constructs of *NbBKI1*. ~300bp fragment of 5'- and 3'- *NbBKI1* were introduced into pTRV2 vector and used for *NbBKI1* silencing. (B) *NbBKI1*-silenced plants showed significantly larger size compared with TRV:EV and TRV:*PDS* controls. (C) *NbBKI1* silencing was detected by semi-quantitative RT-PCR. Both of the 5'- and 3'-*NbBKI1* silencing constructs resulted in reduction of expression level of *NbBKI1* gene. *GAPDH* gene was used as an endogenous reference gene.
