# Supplementary material for: Plasmopara viticola effector PvRXLR131 suppresses plant immunity by targeting plant receptor‐like kinase inhibitor BKI1
Source: Mol Plant Pathol. 2019 Apr 4;20(6):765–83. doi: 10.1111/mpp.12790 (PMC6637860; doi:10.1111/mpp.12790)
Supplement: Supplementary file 11 — Fig. S11 PvRXLR131 suppresses elicitor‐triggered cell death in TRV:EV leaves. (A) Agrobacterium GV3101 carrying pGR106‐PvRXLR131, pGR106‐GFP and pGR106‐PvRXLR21 (positive control) constructs were infiltrated into TRV:EV Nicotiana benthamiana leaves; GV3101 containing pGR106‐INF/BAX was infiltrated at the first infiltration region after 24_h. Cell death was scored 5_days after the second infiltration. The lower line indicates corresponding decolourized N. benthamiana leaves. Quanti?cation of cell death by electrolyte leakage are shown (B). Each data point represents means ± standard deviations (SDs) from three biological replicates; values with different letters show significant differences between mean values at P = 0.01 (LSD). [file MPP-20-765-s011.pdf]

FIGURE S11

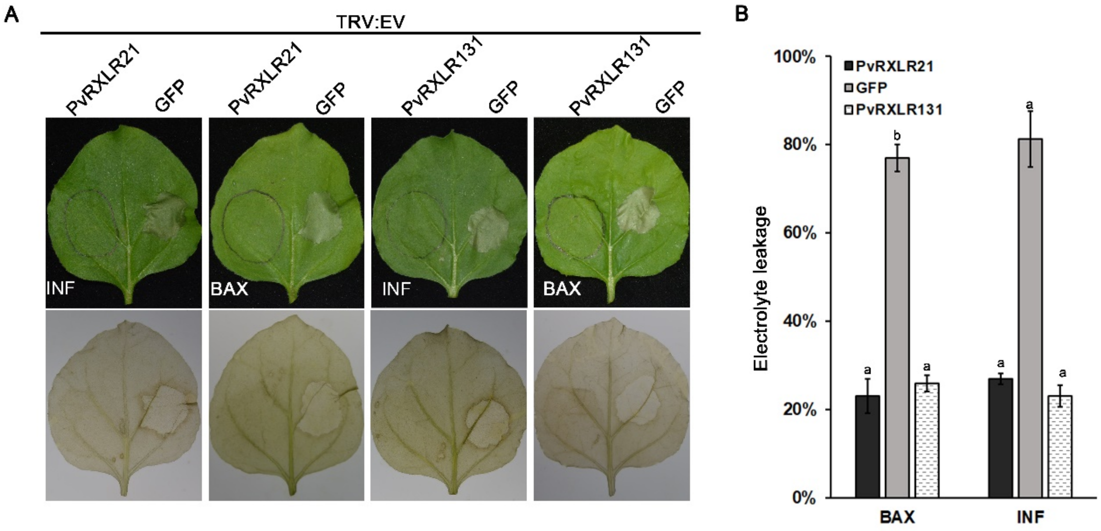

S11 Fig. PvRXLR131 suppresses elicitor-triggered cell death in TRV:EV leaves. (A) *Agrobacterium* GV3101 carrying pGR106-PvRXLR131, pGR106-GFP, and pGR106-PvRXLR21 (positive control) constructs were infiltrated into TRV:EV *Nicotiana benthamiana* leaves; GV3101 containing pGR106-INF/BAX was infiltrated at the first infiltration region after 24 h. Cell death was scored 5 days after the second infiltration. The lower line indicates corresponding decolorized *N. benthamiana* leaves. Quantification of cell death by electrolyte leakage are shown (B). Each data point represents means ± SD from three biological replicates; values with different letters show significant differences between mean values at $p = 0.01$ (LSD).
