# Supplementary material for: Plasmopara viticola effector PvRXLR131 suppresses plant immunity by targeting plant receptor‐like kinase inhibitor BKI1
Source: Mol Plant Pathol. 2019 Apr 4;20(6):765–83. doi: 10.1111/mpp.12790 (PMC6637860; doi:10.1111/mpp.12790)
Supplement: Supplementary file 12 — Fig. S12 Detection of protein expression. (A) Immunoblot analysis of proteins from TRV:EV (EV) and NbBKI1‐silenced (BKI1si) Nicotiana benthamiana transiently expressing PvRXLR131‐Flag, PvRXLR21‐Flag and GFP from the pGR106 vector. (B) Immunoblot analysis of proteins from N. benthamiana leaves transiently expressing GFP and PvRXLR131‐GFP from the pBI121 vector. Ponceau‐S (PS) stained RuBisCO large subunit (rbcL) serves as a loading control. [file MPP-20-765-s012.pdf]

FIGURE S12

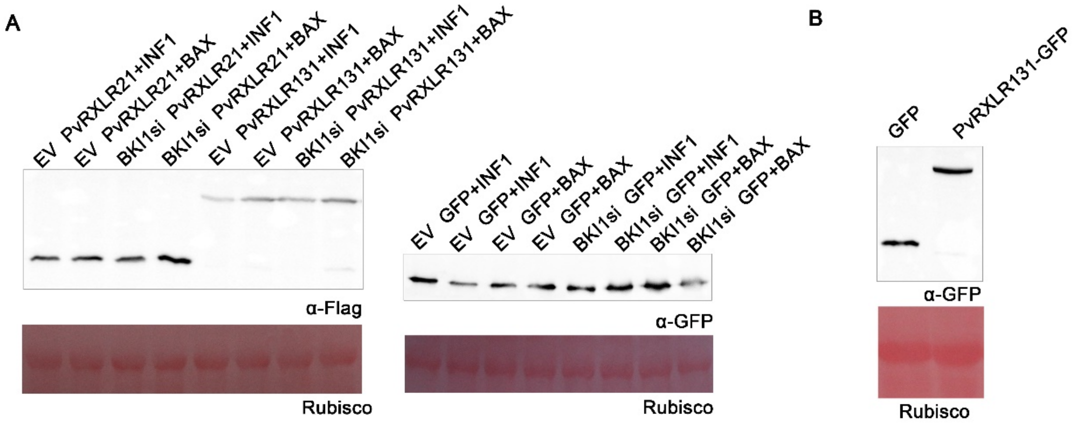

S12 Fig. Detection of protein expression. (A) Immunoblot analysis of proteins from TRV:EV (EV) and *NbBKI1*-silenced (BKI1si) *Nicotiana benthamiana* transiently expressing PvRXLR131-Flag, PvRXLR21-Flag, and GFP from the pGR106 vector. (B) Immunoblot analysis of proteins from *N. benthamiana* leaves transiently expressing GFP and PvRXLR131-GFP from the pBI121 vector. Ponceau-S (PS) stained rubisco large subunit serves as a loading control.
