# Supplementary material for: Plasmopara viticola effector PvRXLR131 suppresses plant immunity by targeting plant receptor‐like kinase inhibitor BKI1
Source: Mol Plant Pathol. 2019 Apr 4;20(6):765–83. doi: 10.1111/mpp.12790 (PMC6637860; doi:10.1111/mpp.12790)
Supplement: Supplementary file 13 — Table S1 Primers for PvRXLR131 plasmid construction. [file MPP-20-765-s013.pdf]

Table S1: Primers for PvRXLR131 plasmid construction

Plasmid	Primer	sequence
pLB	PvRXLR131Full-length-F	ATGCGTCAAATTCCTCTTGTCG
	PvRXLR131Full-length-R	CTACTCGTGAATATACTGATTCGAG
pSUC2	PvRXLR131SP EcoRI-F	CCGGAATTC ATGCGTCAAATTCCTCTTGTCG
	PvRXLR131SP XhoI-R	CCGCTCGAG GCCTTGAAGACGTGCTGC
pGR106	PvRXLR131 ClaI-F	CCATCGAT ATGCTAATAAGCGTGACCAACGC
	PvRXLR131 SalI-R	ACGCGTCGAC CTACTCGTGAATATACTGATTCG
pCB1532	PvRXLR131 XhoI-F	CCGCTCGAG ATGCGTCAAATTCCTCTTGTCG
	PvRXLR131 Bgl II-R	GAAGATCT CTCGTGAATATACTGATTCGAG
pBI121	PvRXLR131 XbaI-F	GCTCTAGA ATGCTAATAAGCGTGACCAACGC
	PvRXLR131 KpnI-R	GGGGTACCCC CTCGTGAATATACTGATTCG
pCAMBIA1300	PvRXLR131 XbaI-F	GCTCTAGA ATGCTAATAAGCGTGACCAACGC
	PvRXLR131 BstBI-R	GGTTCGAA CTCGTGAATATACTGATTCG
pXY106(nYFP)	PvRXLR131 BamHI-F	CGGGATCC ATGCTAATAAGCGTGACCAACGC
	PvRXLR131 SalI-R	ACGCGTCGAC CTACTCGTGAATATACTGATTCG
pColdIII	PvRXLR131 BamHI-F	CGGGATCC ATGCTAATAAGCGTGACCAACGC
	PvRXLR131 SalI-R	ACGCGTCGAC CTACTCGTGAATATACTGATTCG
pGBKT7	PvRXLR131 SfiI-F	TCGGCCATTACGGCC ATGCTAATAAGCGTGACCAACGC
	PvRXLR131 SfiI-R	AGGGCCGAGGCGGCC CTACTCGTGAATATACTGATTCG
	PvRXLR131$^{aa21-119}$ SfiI-F	TCGGCCATTACGGCC ATGCTAATAAGCGTGACCAACG
	PvRXLR131$^{aa21-119}$ SfiI-R	AGGGCCATTACGGCC CTAGTACTCAACATTTTTGATCTG
	PvRXLR131$^{aa58-158}$ SfiI-F	TCGGCCATTACGGCC ATGGGGCTTTCAGATATGC
	PvRXLR131$^{aa58-158}$ SfiI-R	AGGGCCATTACGGCC CTACTCGTGAATATACTGATTCG
	PvRXLR131$^{aa120-158}$ SfiI-F	TCGGCCATTACGGCC ATGAAAGGCAATGTCAAG
	PvRXLR131$^{aa120-158}$ SfiI-R	AGGGCCATTACGGCC CTACTCGTGAATATACTGATTCG

Red font indicates corresponding restriction enzyme recognition sites in the primer name. F: forward primer, R: reverse primer.
