# Supplementary material for: Plasmopara viticola effector PvRXLR131 suppresses plant immunity by targeting plant receptor‐like kinase inhibitor BKI1
Source: Mol Plant Pathol. 2019 Apr 4;20(6):765–83. doi: 10.1111/mpp.12790 (PMC6637860; doi:10.1111/mpp.12790)
Supplement: Supplementary file 14 — Table S2 Primers for BKI1 plasmid construction. [file MPP-20-765-s014.pdf]

Table S2: Primers for BKI1 plasmid construction

Plasmid	Primer	sequence
pLB	VvBKI1-F	ATGGATACCGTACAGCAGCAGAAG
	VvBKI1-R	CTAGTCTTTGCATTTGAGTTTCTC
	VvBKI1^{Y211D}-F	AGCCATGTACTGAAGAGGGACGTGAGAATGGTTCGACCC
	VvBKI1^{Y211D}-R	GGGTCGAACCATTCTCACGTCCCTCTTCAGTACATGGCT
	VvBKI1^{Y211F}-F	AGCCATGTACTGAAGAGGTTCGTGAGAATGGTTCGACCC
	VvBKI1^{Y211F}-R	GGGTCGAACCATTCTCACGAACCTCTTCAGTACATGGCT
	AtBKI1-F	ATGGAAACTAATCTACAACAGGT
	AtBKI1-R	TCAAGAATCCTTAACCTTATCATCA
	AtBKI1^{Y211D}-F	GTCACGCCGTCAAGAAGGACATAAGGATGTTGTTTCAG
	AtBKI1^{Y211D}-R	CTGAAACAACATCCTTATGTCCTTCTTGACGGCGTGAC
	AtBKI1^{Y211F}-F	GTCACGCCGTCAAGAAGTTCATAAGGATGTTGTTTCAG
	AtBKI1^{Y211F}-R	CTGAAACAACATCCTTATGAACTTCTTGACGGCGTGAC
	NbBKI1-F	ATGGACAGCCAATTACACCAAAAC
	NbBKI1-R	TTAGTCTTCCATTGAAATAGATTTC
	NbBKI1^{Y199D}-F	GAGTCGAGTTGTGAAGAGGGACATAAGAATGGTTAGACC
	NbBKI1^{Y199D}-R	GGTCTAACCATTCTTATGTCCCTCTTCACAACTCGACTC
	NbBKI1^{Y199F}-F	GAGTCGAGTTGTGAAGAGGTTCATAAGAATGGTTAGACC
	NbBKI1^{Y199F}-R	GGTCTAACCATTCTTATGAACCTCTTCACAACTCGACTC
pGADT7	VvBKI1 XmaI-F	TCCCCCCGGGGG ATGGATACCGTACAGCAGCAGAAG
	VvBKI1 ClaI-R	CCATCGAT CTAGTCTTTGCATTTGAGTTTCTC
	AtBKI1-F	TCCCCCCGGGGG ATGGAAACTAATCTACAACAGGT
	AtBKI1-R	CCATCGAT TCAAGAATCCTTAACCTTATCATCA
	NbBKI1-F	TCCCCCCGGGGG ATGGACAGCCAATTACACCAAAAC
	NbBKI1-R	CCATCGAT TTAGTCTTCCATTGAAATAGATTTC
pBI121	VvBKI1 XbaI-F	GCTCTAGA ATGGATACCGTACAGCAGCAGAAG
	VvBKI1 KpnI-R	GGGGTACCCC GTCTTTGCATTTGAGTTTCTC
pET3α	VvBKI1 BamHI-F	CGGGATCC ATGGATACCGTACAGCAGCAGAAG
	VvBKI1 XhoI-R	CCGCTCGAG GTCTTTGCATTTGAGTTTCTC
pXY104(cYFP)	VvBKI1 BamHI-F	CGGGATCC ATGGATACCGTACAGCAGCAG
	VvBKI1 SalI-R	ACGCGTCGAC GTCTTTGCATTTGAGTTTCTC
pTRV1	5'NbBKI1 VIGS-F	TCCCCCCGGG ATGGACAGCCAATTMCACCAA
	5'NbBKI1 VIGS-R	CGGGGTACC AGGAAGATGGGAAATAAGGTG
	3'NbBKI1 VIGS-F	TCCCCCCGGG AGAATGGTTAGACCTCTTTTA
	3'NbBKI1 VIGS-R	CGGGGTACC TTAGTCTTCCATTGAAATAGAT

Red font indicates corresponding restriction enzyme recognition sites in the primer name. F: forward primer, R: reverse primer.
