# Supplementary material for: Plasmopara viticola effector PvRXLR131 suppresses plant immunity by targeting plant receptor‐like kinase inhibitor BKI1
Source: Mol Plant Pathol. 2019 Apr 4;20(6):765–83. doi: 10.1111/mpp.12790 (PMC6637860; doi:10.1111/mpp.12790)
Supplement: Supplementary file 15 — Table S3 Primers for quantitative Polymerase Chain Reaction (qPCR). [file MPP-20-765-s015.pdf]

Table S3: Primers for quantitative PCR

Gene	primer	sequence
CPD	AtCPD-F	TTACCGCAAAGCCATCCAA
	AtCPD-R	TCATCACCACCACCGTCAAC
DWF4	AtDWF4-F	GTTGGCCATTTCTTGGTGAAA
	AtDWF4-R	TGGCGGTGTACGGTTTAAGAT
Saur-AC1	AtSaur-AC1-F	TTGGGTGCTAAGCAAATTATTCG
	AtSaur-AC1-R	TCTCCTACATAGACCGCCATGA
U-box	AtU-box-F	TGCGCTGCCAGATAATACACTATT
	AtU-box-R	TGCTGCCCAACATCAGGTT
PvRXLR131	PvRXLR131-F	GGCGTTGAAAAAGGACGC
	PvRXLR131-R	TCCAAGTACACTTAGGATAC
VvActin	VvActin-F	AGACAGGATGAGCAAGGAAATC
	VvActin-R	GCCTCCAATCCATACGCTATAC
PvActin	PvActin-F	CGATCTCGTATCTGAATA
	PvActin-R	CTACATCATCTCATCCAT

F: forward primer, R: reverse primer.
