# Supplementary material for: Plasmopara viticola effector PvRXLR131 suppresses plant immunity by targeting plant receptor‐like kinase inhibitor BKI1
Source: Mol Plant Pathol. 2019 Apr 4;20(6):765–83. doi: 10.1111/mpp.12790 (PMC6637860; doi:10.1111/mpp.12790)
Supplement: Supplementary file 16 — Table S4 Other primers used. [file MPP-20-765-s016.pdf]

Table S4: Other primers used

Gene	primer	sequence
PvRXLR90	PvRXLR90 BamHI-F	CGGGATCC ATGACGGAAGTTCAAGGTACACCAGG
	PvRXLR90 SalI-R	ACGCGTCGAC TCAAAGTCGACGACGACGG
VvBAK1	VvBAK1 BamHI-F	CGGGATCC ATGGAGGCAATCTTTCTCTGC
	VvBAK1 SalI-R	ACGCGTCGAC TCTGGGACCGGACAATTCTTC
GAPDH	NbGAPDH-F	ATGGCATCTGACAAGAAGATCAAG
	NbGAPDH -R	TTAAGCAACAGAAGCCATATGGCAG
Actin	CgActin-F	GGTGCTGCTTTCTGGCAAACTATCT
	CgActin-R	TGAACTCCATCTCGTCCATACCCTC

F: forward primer, R: reverse primer.
